# Communication Requirements in 5G-Enabled Healthcare Applications: Review and Considerations

**DOI:** 10.3390/healthcare10020293

**Published:** 2022-02-02

**Authors:** Haneya Naeem Qureshi, Marvin Manalastas, Aneeqa Ijaz, Ali Imran, Yongkang Liu, Mohamad Omar Al Kalaa

**Affiliations:** 1 Center for Devices and Radiological Health, U.S. Food and Drug Administration, Silver Spring, MD 20993, USA; marvin@ou.edu (M.M.); yongkang.liu@fda.hhs.gov (Y.L.); omar.al-kalaa@fda.hhs.gov (M.O.A.K.); 2 AI4Networks Research Center, School of Electrical & Computer Engineering, University of Oklahoma, Tulsa, OK 74135, USA; aneeqa@ou.edu (A.I.); ali.imran@ou.edu (A.I.)

**Keywords:** 5G networks, healthcare, key performance indicators, wireless communication

## Abstract

Fifth generation (5G) mobile communication technology can enable novel healthcare applications and augment existing ones. However, 5G-enabled healthcare applications demand diverse technical requirements for radio communication. Knowledge of these requirements is important for developers, network providers, and regulatory authorities in the healthcare sector to facilitate safe and effective healthcare. In this paper, we review, identify, describe, and compare the requirements for communication key performance indicators in relevant healthcare use cases, including remote robotic-assisted surgery, connected ambulance, wearable and implantable devices, and service robotics for assisted living, with a focus on quantitative requirements. We also compare 5G-healthcare requirements with the current state of 5G capabilities. Finally, we identify gaps in the existing literature and highlight considerations for this space.

## 1. Introduction

Integrating fifth generation (5G) mobile communication technology into digital healthcare technology can facilitate healthcare delivery with expanded communication capabilities given 5G’s high data speed, ultra-low latency, massive device connectivity, reliability, increased network capacity, and increased availability. These characteristics can enable novel healthcare use cases and augment existing ones [1,2,3,4]. Use cases include remote robotic-assisted surgery, remote diagnosis/teleconsultation, in-ambulance treatment by a remote physician, wearable device applications (wearable device applications are considered within the scope of the Internet of Things (IoT), narrow band IoT (NB-IoT), or Massive IoT), service robotics for assisted living, and medical big data management [1,5,6,7,8,9].

5G-enabled healthcare applications have diverse communication technical requirements for different use cases. Knowledge of those requirements is important for all stakeholders, including developers, network providers, and regulatory authorities in the healthcare sector, to facilitate safe and effective healthcare [6], where an understanding of the underlying communication requirements is needed to select wireless technology with features that support healthcare application design targets and expected performance [10]. 5G promises to provide the low latency and high bandwidth to enable modern healthcare applications such as remote robotic surgery and in-ambulance treatment. Accordingly, designing, deploying, and evaluating the systems needed to implement those use-cases can be informed with a clear understanding of the underlying communication requirements that can enable the intended functionality.

For instance, the expansive set of 5G configuration and optimization parameters offer network operators flexible options in setting up their networks and dynamically optimizing network performance to achieve a desired objective. Accordingly, a large set of parameters can impact the needed performance for a 5G-healthcare use case. Accordingly, quantitative key performance indicators (KPIs) can help 5G network providers assess the feasibility of a given 5G-enabled healthcare use case, provide the level of service needed for the safe and effective functioning of 5G-enabled healthcare applications, and draft service level agreements with their customers. Clearly specified KPIs can also inform regulatory authorities like the U.S. Food and Drug Administration (FDA) when evaluating whether communication service levels and quality of service are met to support the safe and effective use of a 5G-enabled medical device. Finally, end users such as healthcare facilities and patients can use this knowledge for developing, negotiating, and managing relevant service level agreements (SLAs) with the 5G network provider [6].

In this review paper, we identify, compare, and summarize the communication requirements for several healthcare use cases that can be enabled by 5G. The focus of this paper is on quantitative requirements. Furthermore, we identify gaps in the existing literature and highlight considerations in this area. Specifically, we survey the technical requirements for remote robotic-assisted surgery, mobile connected ambulance (i.e., in-ambulance treatment by remote physicians), wearable and implantable devices, and service robotics for assisted living.

This article is unique in detailing a comprehensive review of the quantitative KPI requirements of 5G-healthcare use cases. To the best of our knowledge, the closest work to our review paper on the similar topic is the recent magazine article by Cisotto et al. [11], which highlights select quantitative requirements for the use cases of telepresence and robotic-assisted telesurgery, remote pervasive monitoring, healthcare in rural areas, and mobile health (m-Health). Compared to the related work, our review paper includes references specific to the use of 5G in healthcare, in addition to those addressing the communication requirements of the healthcare applications regardless of the enabling communication technology, which can inform how applications use 5G. Our literature search results extend until 29 June 2021. Accordingly, we have significantly expanded the scope of the considered references to comprehensively capture the state-of-the-art and include a comparative study between planned and existing 5G capabilities. We have also identified gaps in this space and considerations for 5G-healthcare requirements, which were not within the scope of [11]. Moreover, after identifying literature that reported KPIs for the use of 5G in healthcare use cases, we have traced the original sources of the referenced KPIs in those papers.

**Figure 1:**
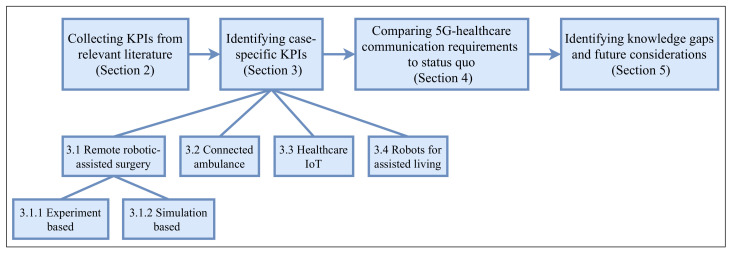
Methodology and organization of paper.

The rest of the paper is organized as shown in Figure 1. In Section 2, an overview of 5G KPIs with specifications of their definitions is provided. Section 3.1, Section 3.2, Section 3.3 and Section 3.4 identify four potential areas of 5G healthcare applications and review KPI requirements in individual areas, which include telesurgery (Section 3.1), connected ambulance (Section 3.2), healthcare IoT (Section 3.3), and robots for assisted living (Section 3.4). The identified 5G-healthcare requirements are then compared with the current state of 5G capabilities in Section 4, and gaps in this space are highlighted in Section 5. Finally, Section 6 concludes the paper.

## 2. Key Performance Indicators for 5G-Healthcare

**Figure 2:**
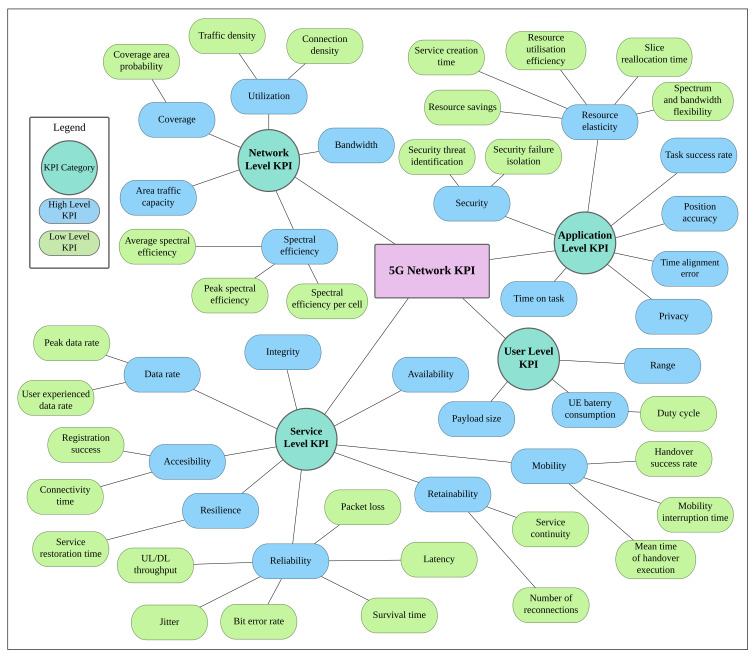
Taxonomy of 5G network KPIs.

While KPIs such as data rate, accessibility, reliability, and mobility have been widely used in the performance evaluation of 4G cellular networks, the diversity and heterogeneity of 5G applications are calling for further expansion to incorporating novel sets of KPIs for measuring adequacy and efficacy of 5G-enabled services. The taxonomy shown in Figure 2 highlights the vastness of 5G network KPIs. Inspired by [12,13,14] and combined with domain knowledge, this taxonomy classifies 5G KPIs into four categories: network, service, application, and user. Each category also includes high-level and low-level KPIs. High-level ones measure the overall performance of the network based on metrics defined by the standardization bodies such as 3rd Generation Partnership Project (3GPP). However, most of the time, these high-level KPIs are focused on characterizing general features of the cellular system/service. In this regard, we also introduce low-level KPIs under each high-level one to further instantiate specific requirements. A certain 5G-enabled healthcare application might depend on a given set of KPIs to deliver its function while having low sensitivity to others.

The service level KPIs often discussed in 5G-enabled healthcare literature to address several aspects of the communication network, including *availability*, *accessibility*, *reliability*, *data rate*, and *retainability*. Availability is the fraction of time the network is available to provide the services users demand [15]. Accessibility is discussed in the context of connectivity time, which measures the time to establish a network connection, starting at the user request and ending at the beginning of the data transmission. Reliability is addressed through numerous low-level KPIs shown in Figure 2: *throughput*, *latency*, *jitter*, and *packet loss rate (PLR)*, and *bit error rate (BER)*. User throughput during active time is the size of a burst divided by the time between the arrival of the first packet and the reception of the last packet of the burst. Latency corresponds to the travel time of data packets from the source to the destination (i.e., one-way, or end-to-end latency) [16]. The round-trip latency is the time it takes a signal to be sent plus the time spent to receive an acknowledgement of that signal. Jitter is a measure of the variation in the time of arrival between packets. If uncontrolled, jitter impacts the audio and video quality, which can negatively impact applications where this type of communication is used (e.g., telesurgery, remote diagnosis, and service robotics for assisted living). PLR is the fraction of packets that failed to reach the receiver out of total number of transmitted packets. BER is the total number of bits received in error over the total number of bits sent. Like jitter, high BER/PLR negatively impacts audio and video quality. Also relevant to the service level is the data rate, which is a measure of the volume of successfully received application data, expressed in bits, within a period expressed in seconds. A high data rate is relevant in applications that transport large volumes of data. Service retainability refers to the count of radio link interruptions following the activation of that link between the user and the network. A related measure of service retainability is the number of reconnections, i.e., the count of attempts a user performs to re-establish network connection following a link failure.

The overall network characteristics are addressed in the literature with several network level KPIs such as network *bandwidth*, *utilization* and *spectral efficiency*. Bandwidth refers to the network maximum aggregated data transmission rate. *Connection density* and *traffic density* are measures of utilization. Connection density refers to the number of connected devices per unit area. This is relevant in connected IoT application, where the number of connected devices is large. Traffic density (or area traffic capacity) is a measure of the volume of catered data in a unit area. Spectral efficiency is the maximum number of bits the network can provide to users every second using a given bandwidth.

On the user level, KPIs of *battery or power consumption*, *range*, and *payload size* are commonly reported in literature covered in this paper. User battery consumption and the its associated low-level KPI, *duty cycle*, which is the ratio between an application active (ON) and idle (OFF) times, are relevant in IoT devices where transmissions are intermittent and battery lifetime is limited. Range is the distance at which the signal transmitted is sufficient for the transmitter and receiver to communicate effectively. Another relevant KPI discussed in literature is the user payload size, which can be controlled to balance the transferred data volume with the incurred transmission overhead. This promotes efficient network resource usage while helping to meet specific application needs.

On the application level, *security* and *position accuracy* are the most commonly discussed KPIs in literature reviewed in this paper. Security refers to the network ability to identify, isolate, and eliminate threats to its infrastructure, users, and their data. Position accuracy is a measure of the difference between the estimated and actual user locations. The 3GPP (the entity that develops 5G specifications) has set different position accuracy targets for different scenarios ranging from several meters for emergency calls to a few decimeters for indoor plant operations and vehicle-to-everything (V2X) [17].

Although relevant to enabling 5G healthcare functions, some KPIs are seldom addressed in the articles reviewed in this paper. For example, the *network-coverage* is relevant to all applications using its services. While network *coverage area probability* is related to user activity range, it refers to the percentage of service area where users can receive a desired service. On the application level, *privacy* is relevant to healthcare applications because it refers to the ability of the network to keep the data that passes through it or is stored privately in it. Also on the application level, network *resource elasticity* is relevant in applications with temporary need for elevated connection capabilities such as in-ambulance treatment and other emergency related applications. Resource elasticity describes the network ability of responding to temporal and spatial fluctuations in traffic demand by redistributing available resources to seamlessly meet the demand of critical applications [18]. On the service level, *mobility* is relevant to applications that are mobile such as connected ambulance. Mobility is the maximum user speed that a network can support. It also refers to the ability of a network to support mobile users. A measure of mobility can be the *rate of successful handovers* between the coverage sites. Additional examples of KPIs related to the service level include the *service restoration time* under resilience and *survival time* under reliability. The former refers to the period in which the services are restored to normal operating status after experiencing a downtime. The latter is the tolerable packet delay in which an application can still function effectively.

**Figure 3:**
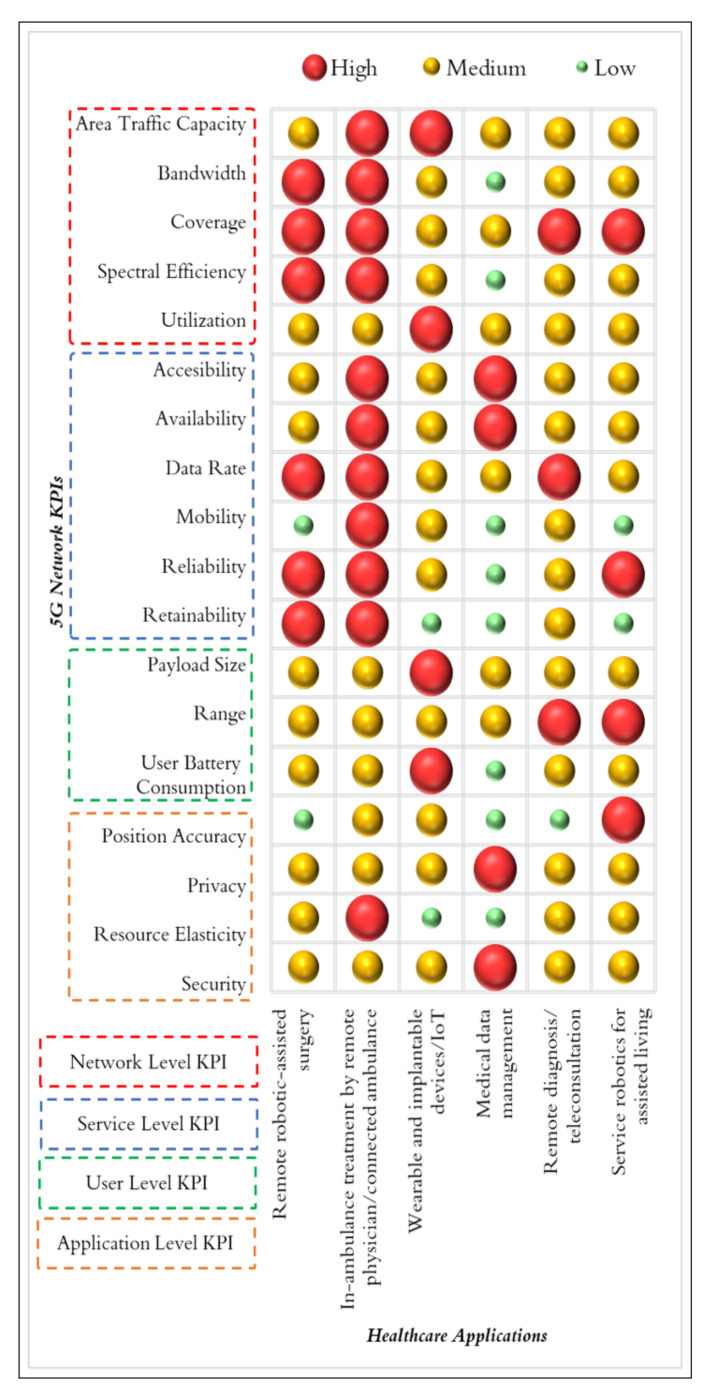
Examples of 5G-enabled healthcare application concepts and their projected needs for some communication KPIs.

Figure 3 illustrates a subjective summary of the general relevance of the high-level 5G network KPIs we investigated in Figure 2 to the following applications: remote robotic-assisted surgery, connected ambulance or in-ambulance treatment by remote physician, healthcare IoT applications, medical data management, teleconsultation and remote diagnosis, and service robotics for assisted living. These applications are only considered as generic concepts, which recognizes that realistic medical devices implementing one or more of these application concepts have unique KPI needs. Furthermore, the FDA guidance document on radio frequency wireless technology in medical devices recommends that the medical device wireless quality of service (QoS) is specific to the medical device [10]. Accordingly, this summary can help inform the KPI value specifications that should be determined for the specific intended use of a medical device and its design. Relevance is qualitatively described as high, medium, or low. Notably, remote robotic-assisted surgery needs careful provisioning of several KPIs, including reliability, where low-level KPIs such as latency, jitter, and packet loss fall under. However, when the scenario is implemented in an operating room, mobility is not as relevant as other KPIs since the connection will not move across multiple network cells. On the contrary, in-ambulance treatment by remote physician or connected ambulance needs exceptional mobility support since the data exchange occurs while the ambulance is mobile. Support for mobility in this case complements other relevant KPIs such as reliability, data rate, availability, coverage, and resource elasticity to enable the exchange of diverse data streams (e.g., video, audio, file transfer, and control commands). The number of connected wearable devices is expected to grow globally from 720 million in 2019 to more than 1 billion in 2022 [19]. Accordingly, the KPIs of utilization and UE battery consumption are highly relevant for enabling the network connectivity for such devices given their energy constraints. In the case of medical data management, security and privacy are more relevant compared to other KPIs, such as reliability. Like other services that use audio and video, remote diagnosis or teleconsultation are negatively impacted with degraded reliability. Other relevant KPIs for this use case include coverage, range, and utilization, to facilitate the service access by many users. Finally, we note that reliability, range, and position accuracy are relevant in the service robotics for assisted living use case where the robot is mobile in a limited area. The following sections will review the related literature for each of these use cases.

## 3. KPIs for Specific 5G-Healthcare Use Cases

### 3.1. Remote Robotic-Assisted Surgery

Several studies have addressed quantitative KPI requirements for remote robotic-assisted surgery, which we also refer to as telesurgery for the remainder of this review. This use case involves the use of a robotic-assisted surgery platform by a surgeon located in a remote geographic location. The most commonly reported KPIs include latency, data rate, and packet loss [11,20,21,22,23,24,25,26,27,28,29,30,31,32,33,34,35,36,37,38,39,40,41,42,43,44,45,46,47]. Few studies have also reported quantitative requirements for reliability, communication service availability, payload size, traffic density, connection density, service area dimension, survival time, range, and duty cycle [11,30,34,44]. Table A1 presents the reported latency requirements for several communication streams that can be used during telesurgery such as camera flows, vital signs, and feedback for force and vibration. Latency in this context is considered end-to-end. Compared to latency, quantitative requirements for jitter have been investigated less investigated in the literature. The reported jitter requirements are detailed in Table A2. Similarly, requirements for data rate are detailed in Table A3. These requirements can be influenced by different compression techniques used. Reported packet loss and BER requirements are presented in Table A4. Reports of other KPIs, such as reliability, availability, survival time, etc., are listed in Table A5. By big payload in Table A5, we mean when the packet exceeds 10 Kb [11]. The ability of current 5G networks to meet these KPIs will be discussed in Section 4.

Notably, the reported KPI values are inconsistent across literature reports, which could be attributed to the varying type of tasks considered by the researchers during telesurgery. Additionally, the equipment used to perform telesurgery and the simulation environment also varies across studies. To detail the context of the telesurgery KPI specification, we also labeled the original source of the reported KPIs in each study as detailed in Table A1, Table A2, Table A3, Table A4 and Table A5. Most KPI values were found in experiment and simulation settings of the individual studies with exceptions where the values were a consensus view of the achievable performance by wireless stakeholders [22,33], and Refs. [22,30] contain a white paper by the 5G Infrastructure Public Private Partnership (5GPPP) that highlighted use cases for 5G in healthcare and suggested quantitative requirements. A technical requirements document was compiled by the IEEE 802.15 Task Group 6 for Body Area Networks (BAN), formed in 2007 to help develop a communication standard optimized for the low power devices and operation, in or around the human body to serve a variety of applications, including medical applications. The report in [30] outlined findings from the National Science Foundation (NSF)-funded workshop on ultra-low latency wireless networks. The report addressed healthcare application requirements of the emerging applications, including telesurgery, in terms of throughput, latency, and reliability. In the following, the relevant experimental and simulation studies are summarized.

#### 3.1.1. Experiment Based

The Aesop 1000TS robot (Computer Motion, Goleta, CA, USA) was adapted to hold a metal pin in addition to a laparoscope and camera (Stryker Instruments, San Jose, CA, USA) in [23]. Programmed incremental time delays were introduced in the audiovisual acquisition, and the number of errors made while performing tasks at various time delay intervals was noted. A remote surgeon in Baltimore, MD performed tasks 9000 miles away in Singapore and determined that a delay of <700 ms is acceptable.

A teleoperation capable ZEUSTM robotic minimally invasive surgery system was used in [24], with a dedicated communication link by Bell Canada and Telesat Canada. This link included a wired link with a roundtrip delay of 64 ms, a satellite link with a roundtrip delay of 580 ms, and a software simulated delay link through a local switch. Different tasks were performed from London, Ontario to Halifax, Nova Scotia, Canada. These included dry (typical surgical maneuvers at latencies from 0 to 1 s, in increments of 100 ms) and wet (internal mammary artery takedown on a pig) experiments. A heuristic mathematical model accompanied the task completion times and error rate results, showing acceptable delays of up to 300 ms and 800 ms for simple tasks with training. It was concluded that the effect of delay is not pronounced until the round-trip time exceeds 400 ms and the maximum tolerable delay is approximately 600 ms.

Researchers from European Institute of Telesurgery used the ZEUS system, which is transcontinental, which attempted a remote robot-assisted laparoscopic cholecystectomy on a 68-year-old woman with a history of abdominal pain and cholelithiasis. The surgeon’s subsystem (Equant’s point of presence, New York) and patient’s subsystem (operating room in European Institute of Telesurgery, Strasbourg) were connected via a high-speed terrestrial network (i.e., ATM service), with a round-trip distance of over 14,000 km. Robot motion data had a high priority and a rate guarantee of 512 Kbps within the 10 Mbps virtual path. The operation was carried out successfully in 54 min, with a 155 ms mean time lag for transmission. The study estimated that 300 ms was the maximum time tolerable delay.

Dohler et al. [32] attempted a robot-assisted laparoscopic gall bladder removal for six pigs, with the surgeon located in Strasbourg, France and animals located in Paris, France, using the ZEUS system. The time lag was artificially increased from 20 ms up to 551.5 ms. It was concluded that no packets were lost during the surgical procedures, and the round-trip delay was 78–80 ms, with additional 70 ms for video coding and decoding and a few milliseconds for rate adaptation, summing to 155 ms [32].

To study the impact of haptic feedback in virtual environments, two experimental platforms were implemented in [40]. Platform 1 consisted of two sites at the University of Belfast separated by a few hundred meters and linked by Gigabit Ethernet connection. The configuration of the experimental platform consisted of four 100 Mbps Ethernet segments, two 1000 Mbps fiber optic segments, and four PCs. One PC was connected to a PHANToM Desktop, two generated background traffic, and one ran the remote virtual environment. In Platform 2, one of the computers is used to emulate network impairments. Haptic data, network congestion, and network-impairments were analyzed using these two platforms by introducing controlled delay (0 ms to 50 ms), jitter (1 ms to 15 ms), and packet loss (0.1% to 50%). Study participants self-scored the sense of force feedback. The haptic QoS requirements were summarized by less than 10 ms delay, less than 3 ms jitter, 1% to 5% for packet loss rate, and haptic data transmission rate of approximately 1 kHz.

The study in [29] involved both simulation and practical experiments, where multimodal data were transmitted over a QoS-enabled Internet Protocol (IP) network. The force feedback device was the PHANToM desktop from SensAble Technologies Inc., which could provide force up to 3.3 N in 3 axis directions and generate 1000 packets/s of position and force data during the haptic collaboration actions. In the experiments, the force feedback device was used to manipulate moving virtual objects and to provide the user with feedback from the virtual environment. The end-to-end delay experienced by the haptic traffic was found to decrease from 200 ms (best effort) to 40 ms by running the haptic application in a Differentiated Services (DiffServ) network.

To understand the impact of vibration feedback latency, authors of [37] built a system consisting of a liquid crystal display (LCD), touch sensor, rod device with a vibrator, microcontroller, and a host computer. The microcontroller (NXP semiconductors, mbed NXP LPC1768) controlled the feedback latency from 0.1 to 25.6 ms, according to an adaptive staircase algorithm. Twenty-four participants first sat in front of the touchscreen and were instructed to tap the touchscreen by raising the rod as quickly as possible after the rod head made contact with the touchscreen with an approach velocity of 0.1–0.5 m/s. After the practice, they experienced a 25.6 ms delayed vibration. The participants then conducted eight staircases for further experiments involving two surface conditions (wood or metal). The results showed a 5.5 ms detection threshold of the vibration feedback latency.

Another experimental study proposed a multiplexing scheme that was evaluated using a teleoperation system consisting of a KUKA light weight robot arm (KUKA Robotics), a JR3 force/torque sensor, a force dimension Omega 6 haptic device [31], and real-time Linux-based Xenomai development software. Using the robot arm, the human operator could move toys and peg them in corresponding holes, which was considered as a representative task for the teleoperation applications. Haptic teleoperation experiments were performed, and KPIs considered were varying end-to-end signal latencies (force delay, video delay, audio delay), packet rates, peak delay, convergence time, and peak signal-to-noise ratio (PSNR) for visual quality.

In [39], researchers from Touch Lab, MIT demonstrated an experiment on haptic interaction between two users over a network with 2.4 Gbps connection. Authors used two PHANToM force-feedback devices at both sites; one was located at UCL VECG Lab, London, UK and the second was in MIT Touch Lab, Massachusetts, USA. The experimental subjects were to cooperate in lifting a virtual box together under different conditions.

A mutual tele-environment system named “HaptoClone” is proposed by researchers from the University of Tokyo in [36], which mutually copies adjacent 3D environments optically and physically using micro-mirror array plates technology. Haptic feedback was also given by using an airborne ultrasound tactile display. Different objects were touched by users, and the perceived delay of tactile feedback was measured. Simulations showed that a 100 ms delay was allowable to achieve the real-time interaction.

Other experimental studies using robot systems of SoloAssist (AKTORmed) in Germany, Panda robot (Franka Emika) in Italy, 3D-microscope (Karl Storz) and TiRobot system (Tinavi), and MicroHand (WEGO Group) in China are surveyed in [46].

#### 3.1.2. Simulation Based

The surgical simulator dV-Trainer from Mimic technologies Inc., Seattle, WA, USA was used in [26,27]. In [26], sixteen medical students performed an energy dissection and a needle-driving exercise on the dV-Trainer, with latencies varying between 0 and 1000 ms with a 100 ms interval. These latencies were communication latencies from the time that a movement was initiated by the surgeon until the image of the movement is visible on the surgeon’s monitor. The difficulty, security, precision, and fluidity of manipulation were self-scored by subjects. It was concluded that the surgical performance deteriorates in an exponential way as the latency increases. This study further concluded that latencies less than 200 ms were ideal for telesurgery; 300 ms was also suitable; 400–500 ms may be acceptable; and 600–700 ms was only acceptable for low-risk and simple procedures. Surgery was quite difficult at 800–1000 ms. The same simulator was utilized in [27]. However, in this study, instead of students, 37 surgeons were involved and performed different exercises in an easy-to-difficult order. The dV-Trainer simulator was permitted to introduce fixed latencies into the exercises between the gesture on the grips and the visual feedback on the console. Instead of a self-scoring system as in [26], the dV-trainer in [27] included a built-in scoring system, capturing instrument collisions, drops, etc. This study concluded that although the impact of delay is related to the difficulty of the procedures, overall, delays of 100 to 200 ms caused no significant impact, delays higher than 500 ms caused a noticeable increase in surgical risk, and surgery became extremely difficult and should be avoided at delays higher than 700 ms.

In [29], following experiments on a testbed (PHANToM devices), a probability density function (PDF) model of the haptic traffic from a distributed haptic virtual environments (DHVE) application was created for the use in a simulated DiffServ network using OPNET simulation tool. Subsequently, the effect of running the haptic traffic over a DiffServ IP network was obtained. Results indicated that the haptic throughput increases with the increase in the queue scheduling weight.

Another work leveraging a similar testbed used a force-feedback haptic device in the PHANToM experimental testbed [41]. The set-up involved two computers that were connected through a gigabit Ethernet fiber optic link running on the best effort IP service. The collected network traces from the test network were used to generate statistical models of each type of DVHE traffic that can be used in the standard network simulation packages such as OPNET. The measured network parameters included throughput, packet lost, delay, and jitter. Results from this simulation model showed a close match of simulation network throughputs with experimental throughputs of 850 Kbps and 630 Kbps in asynchronous and synchronous modes, respectively. DHVE effective throughput deteriorated sharply above 90% background load. End-to-end delays of more than 5 ms occurred at above 90% background load. The impact of jitter, latency, and packet loss was studied in [38] using the analytical models, OPNETWORK, and OPNET simulators. For audio, the simulated traffic behavior model was based on two-state (ON-OFF) Markov modulated rate process (MMRP) with the exponentially distributed time at each state. For video, the model was based on K-state MMRP. The QoS requirements for the audio were reported as: delay < 150 ms, jitter < 30 ms, and packet loss < 1%. For video, these requirements were concluded as: delay < 400 ms, jitter < 30 ms, and packet loss < 1%.

Another simulation-based study to investigate the haptic-audio-visual data communication used an interpersonal communication system, HugMe, which consisted of a haptic jacket for a remote person to simulate nurture touching, a haptic device for a local person to communicate his feelings with the remote person, and a depth camera to capture the image and depth information of the remote person and send it back [28].

Several studies citing jitter requirements for telesurgery have referred to the work in [43] that used Image Server and Haptic Handshake applications. The network emulation in [43] consisted of two endpoint computers and a third intervening computer that simulates the network using NISTNet software. The Handshake application is intended to train students remotely in surgical procedures by placing a haptic device at each endpoint and having the instructor guide the movements of the student remotely. The performance was evaluated under varying packet loss, delay, and jitter conditions. Minimum end-to-end performance requirements for throughput was 128 Kbps, packet loss was less than 10%, delay was less than 20 ms with abrupt movement and less than 80 ms with gentle movement, and jitter was less than 1 ms.

The authors investigated the effect of packet loss and latency in multimodal telepresence systems in [35]. The packet loss caused the impression of time delay and influenced the perception of the subsequent events. The simulated haptic feedback force was generated via PHANToM haptic device. The visual 3D environment was presented on a monitor, which was fixed above the haptic device and tilted $80^{\circ }$ toward the observer. The visual space was collocated with (i.e., projected into) the haptic space by means of a mirror, and participants viewed the mirrored image through a pair of shutter glasses for the stereo image presentation. Visual-haptic event judgment was investigated under packet loss rates of 0, 0.1, 0.2, and 0.3, respectively. The minimum required latency for visual-haptic events was concluded to be 50 ms. Finally, telesurgery reports using software-defined networking (SDN), fog, and cloud infrastructures are described and compared in [48]. For more details on the use of SDN, fog, and cloud in emerging healthcare, the reader is referred to the works in [49,50,51,52,53].

The reported KPI values are inconsistent across literature reports due to factors such as varying types of tasks during telesurgery, varying equipment, and varying simulation environments across the studies. For example, latency ranges from as low as 1 ms for haptic feedback to as high as 700 ms for camera flow data, jitter ranges from 1 ms for haptic feedback to 55 ms for 3D camera flow, and the data rate requirements vary between 10 Kbps for vital signs transmission and 1.6 Gbps for 3D camera flow. Similarly, the BER also varies between $10^{-10}$ to $10^{-3}$ depending on the data type.

### 3.2. Connected Ambulance

Table 1 summarizes the literature relevant to the connected ambulance use case in terms of the investigated communication KPIs. The literature covers a wide range of applications termed connected ambulance. In essence, this involves providing medical care enroute to a healthcare facility while exchanging relevant data (e.g., imaging, vital signs, audio, and video) with healthcare providers. Requirements for 5G-enabled mobile healthcare in general are discussed in [21], where the authors propose to implement two-way connectivity between ambulances and hospitals across the UK. The KPIs discussed in the paper include the maximum allowed end-to-end latency for different data types (i.e., 150 ms for camera and audio flow, 250 ms for vital signs, and less than 10 ms for force and vibration). Data rate requirements for different data types were also specified, with the highest data rate requirement being 10 Mbps for two-way visual multimedia streaming, followed by haptic feedback, including force and vibration data types with 400 Kbps each, and then audio multimedia stream with a requirement of 200 Kbps. Depending on the required quality and bandwidth constraints, the data rate requirements for audio data can vary between 22 and 200 Kbps. Moreover, different types of vital signs were assigned different data rates, with EEG having the highest requirement of up to 86.4 Kbps [21].

The studies in [11,22] also highlighted some general requirements for this use case, including 10 ms latency, 2 ms jitter, <2 ms survival time, $1-10^{-5}$ service availability, $1-10^{-7}$ reliability, and 0.05 Mbps data rate.

The project “improving treatment with rapid evaluation of acute stroke via mobile telemedicine” (iTREAT) in [71] reported that 93% of connected ambulance cases achieved a minimum 9 min of continuous, and live video transmission with a mean mobile connectivity time of 18 min, and 87.5% of tests achieved bidirectional audio video quality with ratings of 4 out of 5 or higher, excluding one route with poor transmission quality. The transport routes were 20 min to the University of Virginia Medical Center, and 30 test runs were performed. Limitations of this study include manual ratings of the service quality, not explicitly incorporating patient while testing, exclusion of one route with poor coverage conditions, small size of study, and being limited to one region.

Another e-ambulance study used biosensor emulators in a laboratory to mimic biosensor communication behavior and studied KPIs with the varying number of biosensors and payload sizes [68,69,70]. Reported outcomes include an upper bound of 250 ms on latency, and 0.4 Mbps for average overall throughput, and the success ratio of transmitted samples varied between 97.7% and 99.9%.

A connected ambulance use case was investigated in [62] in the context of proposing a video encoding configuration that jointly optimizes the clinical video quality, time-varying bandwidth availability, and heterogeneous device’s performance capabilities. The proposed model estimated structural similarity quality with a median accuracy error of less than 1%, bitrate demands with the deviation error of 10% or less, and encoding frame rate within a 6% margin.

The study in [67] proposed measurement-based requirements for high-definition ultrasound images (uplink rate > 20 Mbps, downlink rate > 5 Mbps, network delay < 80 ms, jitter < 30 ms), 4K video (uplink rate > 20 Mbps, downlink rate > 20 Mbps, network delay < 50 ms, jitter < 20 ms). Reliability was set to 99.99%, and mobility was 0–120 km/h. The measured download rate inside the ambulance, which is a user of a 5G private network, reached 1361.21 Mbps, and upload rate reached 257.52 Mbps.

Handling specific patient conditions was also addressed in the context of connected ambulance, e.g., prehospital stroke evaluation and treatment [76]. A Prehospital Stroke Study at the Universitair Ziekenhuis Brussel investigated the safety, technical feasibility, and reliability of in-ambulance telemedicine [58]. A total of 43 attempts were made to perform a prehospital teleconsultation of neurological and non-neurological conditions (e.g., strokes, trauma, respiratory, gastro-intestinal, acute pain, intoxication, labor, dysglycemia, and vascular disease). The authors concluded that 30 teleconsultations were performed, with success rate of 73.2%. Transient signal loss occurred during 6 teleconsultation sessions (14.6%). The time before the connection was re-established varied from 38 seconds to 5 minutes and 47 seconds. Permanent signal losses occurred in five teleconsultations (12.2%). The success rates for the communication of blood pressure, heart rate, blood oxygen saturation, glycemia, and electronic patient identification were 78.7%, 84.8%, 80.6%, 64.0%, and 84.2%, respectively. Communication of a prehospital report to the in-hospital team had a 94.7% success rate and prenotification of the in-hospital team 90.2%. Most problems were caused by unstable bandwidth of the 3G/4G mobile network; limited high speed broadband access; and software, hardware, or human error. The study’s main limitations include the small sample size, short study duration, and complex observational design. A continuation of this study was carried out in [60], which addressed patients with suspected acute stroke and reported median maximal and average upload speeds as 196 Kbps and 40 Kbps, respectively. The download median maximal speed is reported as 407 Kbps, and average speed is reported 12 Kbps, using 4G. An experimental study evaluated the use of mobile stroke treatment units (MSTUs) to diagnose and treat 100 residents of Cleveland who had an acute onset of stroke-like symptoms [61]. It was concluded that there were six instances of video disconnection, of which five were because of an area of poor wireless reception, and one was due to the compatibility issue of the devices. No video disconnections lasted longer than 60 s. One limitation pointed out by the authors is the small sample size of this study.

TeleBAT system in [54] used an integrated mobile telecommunications system while transporting patients to the University of Maryland hospital via an ambulance. Results showed feasibility of the case, with number of disconnections resulting from coverage holes, or network switching.

Another case study on mobile stroke units (MSU), a11, consisted of a combination of two studies: PrioLTE2 (Reliability of Telemedically Guided Pre-hospital Acute Stroke Care With Prioritized 4G Mobile Network Long-Term Evolution) study and TeDir (TeleDiagnostics in Prehospital Emergency Medicine [Tele-Diagnostik im Rettungsdienst]) study. A remote neurologist rated the audiovisual quality. The authors in [74] reported high inter-rater reliabilities between the onboard and remote neurologists, and 16 out of 18 treatment decisions agreed. Limitations of this study included 12.6% of the teleconsultations not being completed due to the failure of video connection, higher rate of aborted attempts than the previous studies (1% in [61] and 2% in [77]), small number of patients, and inclusion of the data from two separate studies with different assessment metrics.

A prehospital utility of rapid stroke evaluation using in-ambulance telemedicine (PURSUIT) pilot feasibility study was conducted in [59]. Actors performing pre-scripted stroke scenarios of varying stroke severity were used in live acute stroke assessments. It is concluded that 80% of the sessions were conducted without major technological limitations. Reliability of video interpretation was defined by a 90% concordance between the data derived during the real-time sessions and those from the scripted scenarios. A previous pilot study, StrokeNET in Berlin, could not conclude assessments because the audio video was lost in 18 out of 30 scenarios [57].

As for cardiac patients, a study published in 2010 [56] demonstrated the transmission of 12-lead electrocardiography (ECG) in an ambulance driving at 50–100 km/h to the cell phone of the attendant emergency medical technician and then to the hospital and to the cell phones of off-site cardiologists using a 3G network, after going through the hospital ECG-processing server. It was concluded that the ECG can be transmitted successfully at the first attempt in all five trials, except in one remote, mountainous ambulance service area. The average transmission time of an ECG report ranged from 91 to 165 s. Interruption of ambulance ECG transmission occurred in up to 27% of transmissions. Rehman et al. in [55] reported a 1 year study included data from 17 ambulances enroute to Silkeborg Central Hospital (distance ranging from 20–75 km) transmitting 12-lead ECGs and involving 250 patients with the suspected diagnosis of acute myocardial infarction. Results indicated that 86% of prehospital diagnoses were successful. Geographically related transmission problems were the primary reason for failure. Limitations of this study included patient history taking by direct communication between the physician and patient and the lack of a randomized setup.

Mobility is one of the unique features of the connected ambulance use cases and this raises the connectivity issues that can be observed in high-speed moving vehicles (e.g., poor signal quality, multiple handovers, greater occurrences of connection drops, and penetration loss from metallic walls of vehicle). To address these challenges, authors in [63] evaluated data streaming between one ambulance and hospital nodes on the uplink with a small cell inside the ambulance traveling at a speed of 120 km/h. In the simulation scenario, a transceiver was installed on the roof of the ambulance to transmit/receive data to/from the backhaul macrocell network. The small cell installed inside the ambulance made a wireless connection between the paramedics and the small cell access point (SAP). The SAP and the transceiver were connected through a wired network. The PLR value when using the small cell was reduced to 4.8% compared to 14% in case of 10 users trying to connect to the outside macrocell base station. All 10 users were located in the same ambulance. Throughput also improved by a small amount with the small cell. Authors concluded that using small cell inside the ambulance could be particularly useful in high bandwidth congestion scenarios. Another way to help address mobility challenges can be to predict the future location of the ambulance based on its previous locations as reported in [66]. The authors proposed an algorithm, NextSTMove, which is 300% faster than traditional algorithms and achieved accuracies of 75% to 100%.

Among the 5G features that can enable connected ambulances is network slicing, where logical network resources can be provisioned to accommodate specific application demands. A study conducted in network slicing environment using facilities at the 5G Prototyping Lab at Dell EMC facilities Ireland and SliceNet reported an average round trip latency of 296.91 ms from client to core, an average round trip time of 50.68 ms from client to edge, and an average packet loss of 7.2% for the core and 0.1% at the edge [65]. Another study was carried out in [64] using the same experimental tools with the added features like QoS control based on the data plane programmability and low-latency cloud-based mobile edge computing (MEC) platform. Throughput was evaluated for the coordinated and uncoordinated network slicing strategies and ranged from 0 to 18 Mbps. In QoS-aware slicing, average delay of less than 0.05 ms was observed. However, in non-QoS aware slicing, no guarantee of low latency was given for any network transmission.

Another network-slicing system architecture for 5G-enabled ambulance service was tested in the experimental settings with ambulance speed of 30 km/h. Two types of data were considered in this study: video data for remote consultation and uploading of 4.5 GB of computed tomography (CT) image data from an ambulance to a destination hospital affiliated with the Zhengzhou University [73]. For video data, the average downlink speed of 1080 p 30 Hz HD video in the 5G network environment was 4.6 Mbps, compared to 3.5 Mbps with unstable network and packet loss in 4G. For CT data, the upload time was shortened by 33 percent in 5G as compared to 4G and the average latency for 5G was 12.88 ms, compared to 76.85 ms for 4G which was 6 times that of 5G.

Other relevant studies are ongoing by the groups such as PRE-hospital Stroke Treatment Organization’s (PRESTO) [75,78] and EU 5G PPP Trials working group by SliceNET [79,80].

The reported KPI values for connected ambulance use case vary across literature reports with the variation in considered ambulance mobility, which has a range of 0–120 km/h across reports. Accordingly, latency ranges from around 10 ms for haptic feedback to around 250 ms for vital signs transmission. However, one study also reports latency of as low as 0.05 ms using a QoS-aware slicing scheme. Jitter ranges from 2 ms to 30 ms, depending on the data type and survival time remains less than 2 ms. The maximum data rate requirement reported in literature is around 1360 Mbps and the minimum is 22 Kbps, depending on the communication quality and bandwidth constraints. The average packet loss is reported to be in the 0.1% to 7.2% range.

### 3.3. Healthcare IoT

**Figure 4:**
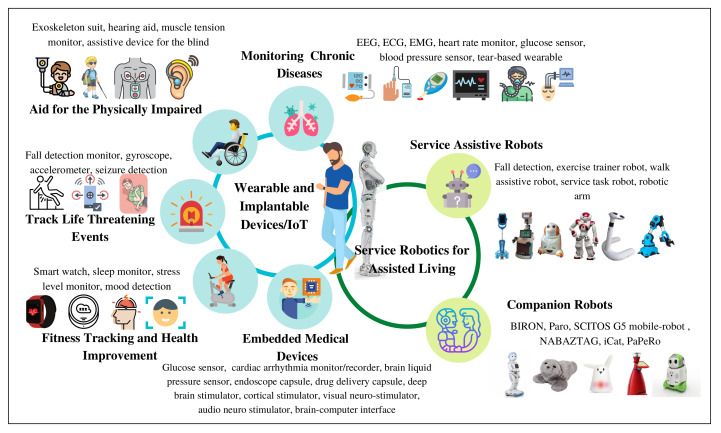
Types ofhealthcare IoT devices and service assistive robots.

Based on the American Society of Engineers, medical internet of things refers to the amalgamation of the medical devices and applications that connect to healthcare information technology systems by leveraging the networking technologies [81]. Healthcare IoT systems encompass diverse applications and computational capabilities and target diverse populations. Notably, many healthcare IoT systems predate 5G and are being used with 4G and local area wireless technologies such as Wi-Fi and Bluetooth. However, 5G can enable an expanded use of healthcare IoT and facilitate the development of novel applications [53]. Accordingly, we dedicate this section to highlighting the wide range of healthcare IoT applications and summarizing their reported communication KPIs. We broadly categorize healthcare IoT systems, which include, medical, and non-medical devices, into five types as shown in Figure 4: (1) fitness tracking and health improvement, (2) chronic disease monitoring, (3) aid for the physically impaired, (4) tracking of life threatening events, and (5) embedded/implantable medical devices.

Applications targeted for healthy individuals can be used for a wide range of purposes, including routine monitoring, lifestyle improvement, or disease prevention, where they act as early warning systems [82]. Examples include smart watches [83,84] that can monitor heart rate, blood glucose level, blood pressure, and breathing rate. Other fitness and health improvement wearables include temperature sensors [85,86]; pulse oximeter SpO${}_{2}$ [87,88,89]; sleep trackers [90]; fertility and pregnancy trackers [91]; and monitors for respiration [92], blood pressure [93,94,95,96], pH [97,98], stress [99], mood [100], and sleep [101].

Patients with underlying conditions or those who need assisted living in chronic scenarios can benefit from applications for measuring and reporting electroencephalogram (EEG) [102,103], ECG [93,104,105], electromyography (EMG) [106,107] heart rate [108,109,110] for cardiac patients, glucose [111,112], insulin for diabetic patients [113,114,115], and continuous respiratory rate for chronic respiratory patients [116]. For assisting the physically impaired, there are numerous wearable devices to help improve quality of life, such as hearing aids (ear-to-ear communication) [117,118]; devices for disability assistance, e.g., muscle tension monitor [119]; muscle tension stimulation [120]; wearable assistive devices for the blind [121,122,123,124]; devices for speech impairment [125,126]; artificial/wearable limbs [127,128,129]; and exoskeleton suits [130]. Other examples that can be used by the elderly, or by Alzheimer’s or epilepsy patients, include wearables for fall detection [131,132,133] and seizure detection [134,135], and gyroscopes [136] and accelerometers [137] for localization monitoring. Examples of implantable devices include pacemakers [138] and implantable cardioverter defibrillators (ICD) [139], and implanted actuator [140,141].

Despite the diversity of healthcare IoT applications, the underlying KPIs requirements are shared by most. However, KPI levels vary for different applications. Following are some of the KPI requirements for this category.

Energy efficiency is vital for battery-operated devices, where the needed battery lifetime can range from a few days to a few years. Accordingly, battery lifetime can be >1 week (the life-time numbers are expected/calculated based on normal use conditions for continuous monitoring) for non-implantable devices, and for monitoring ECG, EEG, EMG, glucose, etc. [142]. For implantable devices, this figure can grow to several years (e.g., >3 years for deep brain stimulator) or remain within the range of hours for some applications such as >24 h for capsule endoscopes [34]. The importance of battery lifetime increases in implanted devices given the risks associated with the device replacement because of depleted battery. In an attempt to overcome constraints on the battery form factor to accommodate specific implant application, solutions for energy harvesting were considered in the literature that can benefit from the energy present in the environment, human body, and wireless signals [143]. Duty cycle is also relevant in this context, where a lower duty cycle contributes to longer battery lifetime. It captures the tradeoff between the need to timely communicate data and the cost of battery power to do so. The work in [34] reports on duty cycle requirements ranging from <1% (e.g., temperature sensors, fall detection devices, and respiration monitors) to <50% (e.g., implantable endoscope capsules).

The efficiency of data transmission during the device ON time is described by the data rate, with varying requirements according to the application and the used transmission protocol. Literature reports offer a wide array of data rate requirements. For example, the researchers in, patel2010applications report that monitoring devices for temperature, heart rate, breathing, blood pressure, blood sugar, and oxygenation require <10 Kbps data rate, 72 Kbps for ECG, 86.4 Kbps for EEG, 1 Mbps for deep brain stimulation and capsule endoscopy, and 1–1.5 Mbps for EMG and location tracking devices [34,144]. Other references [142,145,146,147] listed different values, including 128–320 Kbps for deep brain stimulators, 3 Kbps per ECG channel per link, and 16 bps for the wearable temperature sensors. Data rate can be influenced by device processing capabilities, the data use model (i.e., real-time processing by an external processor is associated with demand for a high data rate, while applications suitable for post-processing can use a low data rate), and the capabilities of the wireless technology being considered. With the advancement of 5G, literature reports now point to a higher data rate to be supported by wearables (e.g., 10 Mbps [148], 0.1–5 Mbps [11].) Requirements for BER also varied by application and were reported in [149], generally ranging from $10^{-10}$ to $10^{-5}$. Specific examples included an ultrasonic wearable device prototype designed to be used as heart rate monitor, and ECG respiratory rate monitor, and step counter reported a BER requirement of lower than $10^{-5}$ using a transmission power of 13 dBm [150]. BER for vital sign monitoring devices such as ECG, pulse oximeters, and implantable devices such as hearing aids are reported as <$10^{-10}$ [34]. To facilitate the diverse healthcare IoT applications, the overall reliability and service availability should be $1-10^{-3}$ [11].

Latency requirements also varied across the applications and by the source. The authors in [144] report <50 ms latency for monitors of chronic disease and emergency event detection. Vital signs monitors were assigned a latency of <1 s, while fitness tracking devices increased latency tolerance to a few seconds. A blanket latency requirement for wearables was set at 250 ms in [11,34], while survival time was set at 10 ms in [11,22] and jitter <25 ms in [11]. Other reported latency values include <50 ms for deep brain stimulators and <100 ms for hearing aids [142]. In [151], LTE-based data transmission experiments using a real-time video wearable device (i.e., BlueEye) under impaired channel loss and propagation loss were performed. The purpose of the study was to test whether mHealth services could be used in the locations with poor coverage conditions. For different mobility scenarios, the jitter values obtained were 0.473 ms for the static users, 2.05 ms for the pedestrian users, and 3.54 ms for the vehicular users. In an attempt to reduce latency in healthcare IoT applications, significant research was dedicated to data processing and analytics at the edge side of the system to circumvent delays caused by the processing lag and cross network data transfer [53,152]. In this context, latency of transmitting various raw ECG captures from a gateway to a remote cloud was compared with the total latency of processing on fog computing service and transmitting preprocessed ECG data in [153]. At the data rate of 9 Mbps there was 48.5% latency reduction by leveraging fog computing in this case. This comes at the cost of addressing data security and privacy while in transport between the device and the cloud. To help manage medical device risks, including security, a risk management process is specified in the international organization for standardization (ISO) 14,971 standard for the application of risk management to the medical devices [154]. Moreover, the FDA published a draft guidance on the content of premarket submissions for the management of cybersecurity in medical devices [155], which provides recommendations to industry regarding cybersecurity aspects of the medical device cybersecurity management, such as risk assessment. Security KPIs in the context of 5G-enabled healthcare applications are summarized in [6], including authenticity, confidentiality, integrity, agility, vulnerability, resilience, mitigation/recovery time, and proactiveness.

Network-level KPIs were addressed in the context of healthcare IoT, including a connection density of 20,000 devices/${\mathrm{km}}^{2}$ in remote pervasive monitoring settings such as in smart home wearables and 10,000 devices/${\mathrm{km}}^{2}$ for general mHealth wearables [11,22]. Other reported KPIs include $50\;{\mathrm{Gbps}/\mathrm{km}}^{2}$ traffic density and 50 km user activity range [11].

Given that the healthcare IoT includes diverse applications that can be used in diverse environments, their enabling KPIs can be influenced by practical deployment factors such as number of nodes, topology, operating frequencies, transmit power restrictions height of device [156], interference, and co-existence [156,157], and others. Finally, we note that one of the emerging 5G-enabled healthcare applications is medical augmented reality/virtual reality (AR/VR). According to a study by Qualcomm [158], the requirements for AR/VR can go to as high as 10–50 Mbps for $360^{\circ }$ 4 K video, 50–200 Mbps for $360^{\circ }$ 8 K video, and up to 5000 Mbps (or 5 Gbps) for 6 degree-of-freedom (DoF) video. Moreover, a study by Facebook indicates a real-time playback rate of 4 Gbps (or 32 Gbps) for 6 DoF video, indicating there might be some use cases where individual sustained per-user rates of >1 Gbps might be needed [159]. The varying applications and diverse IoT device categories contributed to the reported KPI covering a broad range of values. For instance, the battery lifetime ranges from 24 h for capsule endoscopes to several years for other implantable devices. The data transmission rate for wearable devices varies from as low as <10 Kbps to 10 Mbps. Similarly, the BER also varies between $10^{-10}$ and $10^{-3}$ depending on the data type. The latency ranges from 0.473 ms for wearable devices for vital signs monitoring to a few seconds for fitness tracking devices, while the network-level KPIs include a connection density of 10,000–20,000 devices/km${}^{2}$.

### 3.4. Robots for Assisted Living

Robots in assisted living environments have been widely studied in literature [11,20,21,22,23,24,25,26,27,28,29,30,31,32,33,34,35,36,37,38,39,40,41,42,43,44,45,46,47]. An assistive robot can be defined as an aiding device that has the ability to process the sensory information for helping the physically/mentally impaired or elderly persons to perform tasks of daily living without the need of attendants, in hospital or at home [160]. Assistive robots can be broadly classified into two categories, i.e., services assistive robots and companion robots as shown in Figure 4. In this section, our focus is on the communication KPIs for this application with a summary provided in Table 2 of the reported cellular network KPIs.

Position accuracy is pertinent to robots used for fall detection and real-time assistance. The authors in [172] demonstrated that by exploiting the information from the reflected multipath components, increased accuracy and robustness in localization can be achieved. Moreover, they proposed 5G mmWave as one of the promising solutions for indoor accurate localization for assistive living.

According to the EU Horizon 2020 project “Robots in Assisted Living Environments” [173], assisted living considerations include reliability, connectivity, low battery discharge profile, low latency, high communication success rate, and minimum localization error, with appropriate feedback to support people with limited mobility, who require assistance and companionship.

To provide personalized medical support to the elderly in the presence of several chronic diseases, the authors in [167] designed a hybrid robot–cloud approach. The robot autonomously reached the user with the pre-set reminder events acting as a physical reminder. This case study in DomoCasa Lab (Italy) evaluated the robot (DoRo) based on KPIs such as latency (i.e., round trip time), retainability (i.e, in terms of total service time), robot processing time (RPT), average travel time, and mean velocity. Latency over the 20 experimental trials was reported as 56 ms and RPT as 0.012 ms. For the use case where DoRo had to travel 12.6 m to deliver the services with a mean velocity of 0.31 m/s, the total service time was 40.08 s.

The ASTROMOBILE system was evaluated in [169]. The mean path length for the simplest use case (moving in the kitchen) was 9.6 m with a mean velocity of 0.51 m/s, path jerk of 0.023 × $10^{6}$, and a mean position accuracy error is 0.98 m.

Under the German research project SERROGA, which lasted from 2012 to mid 2015, a companion robot for domestic health assistance was developed [164]. Its services include communication, emergency assistant, physical activity motivator, navigation services, pulse rate monitoring, and fall detection. The robot was evaluated in different apartments and labs for a minimum of 29 min and a maximum duration of 255 min, with a velocity range of 0.25–0.27 m/s for distance covered of 355–2600 m. The robot was able to complete the user following tasks with a positioning accuracy of 95%.

A cloud-robotic system for the provisioning of assistive services for the promotion of active and healthy ageing in Italy and Sweden was assessed in [166] on the basis of latency (i.e, round trip time), PLR (i.e, data loss rate), position accuracy (i.e, mean localization error), and localization root mean square error (RMSE) KPIs. The reliability and responsiveness of the cloud Database Management Service (DBMS) was evaluated based on latency as the time a robot waits for the user position, after a request to the server. The study took place in two sites: smart home in Italy (Domocasa lab) and residential condominium in Sweden (Angen). The mean latency in Domocasa lab was 40 ms, while for the Swedish site it was 134.57 ms. The local host latency acquired during the experimentation was 7.46 ms and was used as a benchmark. The rate of service failures was less than 0.5% in Italy, and 0.002% for the Angen site. In Domocasa and Angen, the mean absolute localization errors were 0.98 m and 0.79 m, respectively, while the RMSE were 1.22 m and 0.89 m, respectively. On average, the absolute localization error considering the two setups was 0.89 m, and the RMSE was 1.1 m. The use of the presence sensors increased the localization accuracy in the selected positions by an average of 35%.

Assistive living robots domain can suffer from errors caused by the communication connection issues, latency, and spatiotemporal dynamic environment changes. To improve the autonomy and efficiency of robots in smart environment, the authors in [174] proposed a framework for the improvement of the assistive robot performance through a context acquisition method, an activity recognition process, and a dynamic hierarchical task planner. Additionally, authors in [175] proposed to use full duplex 5G communication for reliable and low-latency robot-based assistive living.

In a trend similar to the other investigated use-cases, the reported communication KPI values for assistive robots varied across reports, with latency varying from 7.46 ms to 134.57 ms and velocity varying from 0.25 m/s to 0.51 m/s. The localization error has a narrow range from 0.89 m–0.98 m, while the distance covered by the assistive robots has a broad range from 12.6 m–2600 m, and service time varies from 0.08 s–255 min.

## 4. 5G-Healthcare Requirements vs. Status of 5G Capabilities

5G technology was developed to meet the use cases specified by the International Telecommunication Union (ITU) International Mobile Telecommunications-2020 (IMT-2020). These are enhanced mobile broadband (eMBB), ultra-reliable, and low-latency communications (URLLC), and massive machine type communications (mMTC). As detailed in the previous sections, many healthcare applications can benefit from the communication capabilities of these 5G use cases. A study based on simulation confirmed that the 3GPP 5G system complies with the ITU IMT-2020 performance requirements [176]. 5G trials and commercial deployments are accelerating throughout the world [177,178,179]. These show varying levels of performance toward theoretical goals. For example, 2 Gbps throughput and 3 ms latency were achieved in Austria using spectrum in the 3.7 GHz band [177]. In another 5G trial in Belgium, 2.94 Gbps throughput and 1.81 ms latency were achieved. The peak throughputs of 15 Gbps, 5 Gbps, and 4.3 Gbps in 5G trials were also reported by European network operators Telia, Elisa, and Tele2 Lithuania, respectively [177]. In the U.S., AT&T reported on 5G use cases such as video streaming, downloading, and conferencing and achieved upload and download speeds around 1 Gbps [177]. Sprint tested streaming 5G virtual reality systems and 4K video and achieved peak download speeds of more than 2 Gbps using the 73 GHz mmWave spectrum [178]. Verizon achieved 4.3 Gbps speeds by aggregating C-band spectrum with mmWave spectrum in a lab trial [179].

Although commercial 5G coverage is still limited [180,181,182], 5G tests by OpenSignal in 2020 compared services offered by Verizon (mmWave), T-Mobile (mmWave, 600 MHz), Sprint (2.5 GHz), and AT&T (850 MHz) [183]. The report concluded that users should not automatically expect speeds of several hundred Mbps on 5G because in the tests they observed an average 5G download speeds ranging from 47.5 Mbps to 722.9 Mbps. They also noted that the U.S. carrier’s 5G services are held back by 5G spectrum availability and some services are fast; however, they are limited by the coverage. Those with greater coverage offer slow speeds due to the limited spectrum. They also highlighted the need for the U.S. carriers to repurpose large portions of the mid-band spectrum for 5G in the U.S. to facilitate the 5G performance goals.

Comparing the realistic performance reports with the most stringent data rate requirement for telesurgery (i.e., 1.6 Gbps for 3D camera flow as listed in Table A3), we note that the throughput requirements of many healthcare use cases might be possible to meet with existing 5G capabilities. However, use cases requiring 6 DoF content such as AR/VR might be challenging those current capabilities. Furthermore, our review highlights that the latency for the haptic feedback can go as low as 1 ms, and for connected ambulance, the lower limit is 10 ms. However, realistic latency figures are expected to remain in the 10–12 ms range [184,185], rather than 1–2 ms. Notably, the 1 ms latency is specified in next-generation radio access network (NG-RAN) domain, which is defined as the link between the end user and base station (including MEC). This latency increases when the communication needs to be transmitted to the core network. Therefore, the end-to-end latency target could be around 5 ms [186]. The additional delay can impact the applications that utilize the core network (e.g., remote expert for collaboration in surgery, video analytics for behavioral recognition, and remote patient monitoring). 5G mmWave frequencies—also known as frequency range 2 (FR2)—can support large subcarrier spacing, resulting in smaller transmission time interval and thus improving latency. This indicates a favorable latency requirement support for healthcare use cases when using the mmWave spectrum. However, this comes at the expense of limited coverage due to the wave propagation properties in the mmWave spectrum, which can impact applications that need mobility support such as the connected ambulance. Moreover, the realistic deployments and trials are limited by the specific used configurations and the small set of reported KPIs like downlink throughput and latency. Accordingly, enabling a specific healthcare application using 5G requires a collaboration between the application developer, 5G network service provider, and the application user to ensure that the service meets the application requirements for communication and that the application can be used safely.

## 5. Gaps in Literature and Future Considerations

A considerable part of the existing literature addresses the communication requirements for the healthcare applications qualitatively, for example, using descriptors such as “big”, “small”, and “extremely low”. Where quantitative requirements are mentioned, the focus is on high-level KPIs, which leaves a gap in describing how a given application can be supported in certain scenarios. For example, when addressing throughput, uplink and downlink throughput are commonly discussed; however, cell edge throughput is not considered. Similarly, mobility is commonly mentioned in terms of speed in the case of connected ambulance, but other mobility-related KPIs, such as handover success/failure rates or handover execution time, are not specified.

Although some reports describe individual KPIs in detail, the trade-offs between multiple KPIs and their interactions with configuration and optimization parameters (COPs) in a healthcare applications are often omitted. For example, one trade-off between throughput and latency for next-generation video content is described in [158], which states that achieving 5–20 ms latency requires 400–600 Mbps throughput, while achieving 1–5 ms latency requires 100–200 Mbps throughput. Another example of trade-offs is between coverage, capacity, and load balancing [187], or the trade-off between coverage, height of BS, and antenna parameters [188]. Such trade-offs are rarely considered in the literature on 5G-enabled healthcare use cases, which can complicate applications with conflicting requirements such as achieving high throughput with high mobility or low battery consumption. One way to study these trade-offs might be to combine several KPIs into a new one. For example, Samsung developed representative KPIs to describe the performance of multi-objective optimization involving more than two KPIs, such as sum of log of data rate, considering both throughput and fairness. It can be used as a joint KPI of wearable devices applications to represent both energy efficiency and throughput, energy efficiency, and delay, or energy efficiency and reliability [189].

Another gap in the literature is the limited 5G network scenarios that are assessed. Limitations include the small number of network trials, small number of infrastructure configurations, small coverage area, and the lack of spatiotemporal variability for trials being conducted in the laboratory settings. A critical analysis of 5G network failure modes that can impact 5G-enabled healthcare use cases is an open question not addressed in the literature. For example, only the success of the connected ambulance use case is discussed in the literature. However, this use case might be negatively impacted in situations with extremely high mobility, high user density, a disaster scenario where a large number of ambulances rush to the same point, a cell outage, or the presence of multiple critical traffic flows in the network.

Moreover, network KPIs are commonly vendor-specific, where each network equipment vendor specifies the performance metrics using its own set of counters and naming conventions. This may give rise to the challenge of managing non-standardized KPIs. The large number of technical counters in the heterogeneous 5G deployments, the use of vendor-specific monitoring tools by the network operators, and the lack of unified data format for collecting and reporting the performance data also pose a challenge for managing the service level agreements between the 5G network operators and the end users of the 5G-enabled healthcare systems [6]. For further reflection on avenues for addressing the highlighted considerations in practice and research, the reader is referred to [6,190].

Finally, we note that real-time systems and time-sensitive networks (TSNs) can benefit several of the discussed healthcare applications such as remote robotic-assisted surgery and in-ambulance treatment. This can be supported by 5G’s technical features such as the near-instantaneous data transmission. For instance, the telerobotic spinal surgeries conducted using 5G-enabled robots have been enabled by a minimal lag between the robot and the remote physician [191]. Similarly, authors in [192] presented a survey on application requiring near real-time response, including healthcare applications such as AR, VR, tele-diagnosis, tele-surgery, and telerehabilitation. Accordingly, future considerations for 5G-enabled healthcare include the investigation and analysis of real-time systems and TSNs and their role in supporting healthcare applications. 5G can also contribute to enabling connected healthcare applications in small-scale healthcare facilities like those in rural areas [193,194].

## 6. Conclusions

5G communication features promise to enable novel healthcare applications and expand network access in the existing connected medical devices. Understanding the communication KPI requirements for 5G-enabled healthcare use cases can help healthcare application developers, 5G network providers, and regulatory authorities in the healthcare sector to promote safe and effective healthcare. In this paper, we have surveyed quantitative and qualitative KPI requirements for different use cases, including remote robotic-assisted surgery, mobile-connected ambulances, wearable and implantable devices in the healthcare IoT, and service robotics for assisted living. A comparison of 5G-healthcare requirements with the status of 5G capabilities reveals that some healthcare applications can be supported by the existing 5G services while others might be challenging, especially those with stringent latency requirement. This calls for a collaboration between the healthcare application developers and the network service providers to explore, document, and manage the possible connectivity support for a given application throughout its lifecycle.

We have also identified gaps in the existing literature and highlight considerations in this space, including the lack of focus on quantitative requirements, omitting relevant KPIs, overlooking the trade-offs between multiple KPIs and COPs, the lack of unified KPI specifications across different network operators and equipment vendors, and (lastly) the limitations 5G scenarios conducted in the existing trials. The gaps in this space and considerations highlighted in this paper can help direct future 5G-enabled medical device studies and facilitate the safe, effective, and efficient implementation of 5G technology in healthcare. Medical devices must integrate 5G technology safely and effectively to facilitate patient access to 5G-enabled medical device applications. As a part of the overall medical device risk management process, documenting and meeting the communication requirements for diverse 5G-healthcare use cases comes under service level agreements. Therefore, knowledge of requirements for 5G-enabled medical use cases highlighted in this paper can also help network service providers, users, and regulatory authorities in developing, managing, monitoring, and evaluating service-level agreements in 5G-enabled medical systems.

## Figures and Tables

**Table 1:** Summary of literature for relevant connected ambulance KPIs.

Use Case	KPIs	Data Type	Tools	Study Year
Ambulance transporting stroke patients to hospital	Throughput, number of reconnections	Audio, video, and vital signs	TeleBAT system in ambulance	[54] 2000
Ambulance transporting cardiac patients to hospital	Retainability, PLR	12-lead ECGs	Rhythm-surveillance and defibrillation equipment	[55] 2002
Ambulance transporting cardiac patients to hospital	Latency, PLR	12-Lead ECG	Philips standard (basic device model without advanced features such as computer-assisted ECG interpretations), embedded, and integrated ECG device	[56] 2010
Ambulance transporting stroke patients to hospital	Retainability	Audio, video	VIMED CAR, head and body cameras, and specialized microphones	[57] 2012
Ambulance transporting stroke patients to hospital	Retainability, bandwidth (mean and maximal upload and download speeds for data transfer), accessibility	Audio-video, blood pressure, heart rate, blood oxygen saturation, glycemia, and electronic patient identification	PreSSUB 3.0 system in ambulance	[58] 2014
Ambulance transporting stroke patients to hospital	Reliability, retainability	Audio, video	In-Touch RP-Xpress telemedicine device, Verizon Jetpack 4G LTE mobile hotspot (4620LE) for 4G LTE	[59] 2014
Ambulance transporting stroke patients to hospital	Bandwidth (median maximal and average upload download speed)	Audio-video, blood pressure, heart rate, blood oxygen saturation, glycemia, temperature, cardiac rhythm, Glasgow Coma Scale (GCS), and electronic patient identification	PreSSUB 3.0 system in ambulance	[60] 2016
Mobile stroke treatment units for patients with acute onset of stroke-like symptoms	Service restoration time, PLR, and latency	CT, audio-video, and vital signs	MSTUs with CT system, camera (RP-Xpress; InTouch Health)	[61] 2016
Testing of video encoding framework on ultrasound videos of carotid artery in connected ambulance scenario	Bitrate, data rate, time-varying bandwidth availability	Ultrasound videos of the common carotid artery	Multi-objective optimization, Philips ATL 5000 ultrasound machine, x265 open source software, and Ubuntu 14.04.4 LTS/Linux 64-bit platform	[62] 2017
A mobile small cell-based ambulance in the uplink direction in a heterogeneous network	Latency, data rate, PLR, retainability, and spectral efficiency	Ultrasound video	LTE Sim system level simulator	[63] 2018
Project proposal aiming to capture more than 6000 ambulances across the UK provided by 200 different vendors	Latency, data rate, PLR	Ultrasound video, in-ambulance video vital signs, EEG, ECG, force, vibration	Sonography and vital-signs-measuring equipment in ambulances	[21] 2019
Connected ambulance prototype study with QoS control in network slicing environment	Uplink/downlink throughput, latency (average per-hop)	Video slices (eHealth, conferencing, surveillance and entertainment)	MEC-based TeleStroke service by SliceNet, NetFPGA cards, SimpleSumeSwitch architecture, LTE eNodeBs, OpenFlow-enabled switches, Software Development Kit (SDK), Dell Edge Gateway, and P4 NetFPGA	[64] 2019
Connected Ambulance prototype study in network slicing environment	Average packet loss, latency (round trip time), throughput (frames per second)	Audio, video	eHealth infrastructure at Dell, Ireland, pfSense security, OpenVPN, Dell Edge Gateway series 3003, LTE SIMS, OpenMANO OSM, and MEC by SliceNET	[65] 2019
Prediction of ambulances’ future locations to overcome mobility-based challanges	Position accuracy	GPS data	Apache Spark, Spark SQL, and algorithms	[66] 2020
Proposition of an architecture for connected ambulance	Uplink/downlink rate, number of device connections, latency, speed, reliability, and jitter	Ultrasound image, vital signs, and video	Vital signs monitor, ultrasound equipment, and video cameras	[67] 2020
Report compiled by industry experts and academic researchers based on their studies	Latency, jitter, survival time, communication service availability, reliability, and data rate	4K video, audio	Reference given to [22]	[11] 2020
Simulation of mobile ambulance using emulated biosensor data	Latency, average throughput, and PLR	Body temperature, blood pressure, and heart rate	Data Distribution Service (DDS) middleware, and biosensor emulator	[68,69,70] 2015, 2020
Ambulance transporting stroke patients in rural area to hospital	Retainability, reliability	Audio, video,	iPad, Jabber video app, University of Virginia Health System firewall, COR IBR600 LE-VZ; CradlePoint router, 4G Verizon Wireless sim, and AP-CW-M-S22-RP2-BL and AP-CG-S22-BL antennas	[71,72] 2016, 2020
Connected ambulance evaluation in network slicing environment using a test platform	Downlink/uplink data rate, and uplink latency	Video, CT image, vital signals, and medical record	5G customer-premises equipment (CPE) signal transceiver, 5G user plane function (UPF) gateway service flow forwarding device, and medical data acquisition device, MEC cloud computing node	[73] 2021
Stroke patients in mobile stroke units en route to hospital	Reliability, retainability	Audio, video, ECG, and vital signs	MEYTEC GmbH telemedicine systems of Vimed car and Vimed Doc for videoconferencing and teleradiology	[74,75] 2019, 2021

**Table 2:** Summary of literature for relevant assistive robots KPIs.

KPI	Service Robot	Assigned Tasks	Target Population	Study
UE battery	Mobile robot BENDER with telepresence capabilities	Assistance in routine tasks and user localization	Elderly	[161]
Latency, PLR	Companion robot	User finding and medication reminder	Elderly	[162]
Latency, data rate	Cloud robot	Monitoring of vital signs	Elderly	[163]
Accessibility, position accuracy	Domestic health assistant Max	Assistance in routine tasks, user searching and following	Healthy elderly	[164]
Throughput (packets per seconds)	Domestic robot DoRo	Video streaming through robot cameras	Elderly and children	[165]
Latency, PLR, position accuracy (mean localization error)	Service robot	Recognition and localization of users	Healthy elderly	[166]
Latency (round trip time), retainability (total service time)	Mobile robot DoRo	Personalized medical support and pre-set reminder event	Elderly people with chronic diseases (multimorbidity)	[167]
Latency, reliability	Nao, Qbo and Hanson robots	Streaming of teleoperation website	Elderly and children	[168]
Position accuracy	ASTRO robot	Assistance in routine tasks, health related reminders	Healthy elderly	[169]
Position accuracy	Assistive robotic arm	Tablet placement infront of patient	Patients with limited or no mobility	[170]
Position accuracy	Mobile humanoid robot GARMI	Support for household tasks and emergency assistance	Elderly and patients	[171]

## Data Availability

Not applicable.

## Appendix A. Telesurgery KPIs

**Table A1:** Latency requirements for telesurgery.

Data Type	Reported Latency	Source	Distance
2D camera flow	<150 ms [20,21]	Experiment [32]	14,000 km
	<200 ms [11,22]	Other [22]	≈1000 m
	<700 ms [23]	Experiment [23]	9000 miles
	<600 ms [24]	Experiment [24]	14,000 km
	<300 ms [25]	Experiment [25]	14,000 km
3D camera flow	<150 ms [20,21]	Experiment [32]	14,000 km
	<300 ms [26]	Experiment [26]	-
	<500 ms [27]	Experiment [27]	-
	<400 ms [28,29]	Simulation [38]	-
	280 ms [195]	Experiment [195]	15 km
	20–50 ms [30]	Other [30]	200 km
	2–60 ms [46]	Experiment [196]	-
	146–202 ms [197]	Experiment [197]	4 km, 6.1 km
	28 ms [191]	Experiment [191]	≈740 km, 1260 km, 144 km, 190 km, 3160 km
	258–278 ms [198]	Experiment [198]	3000 km
Audio flow	0.25–5 ms [48]	Simulation [48]	-
	<150 ms [20,21,28,31]	Experiment [32]	14,000 km
	100 ms [30]	Other [30]	200 km
Temperature	<250 ms [11,20,21,33,34]	Other [33]	-
Blood pressure	<250 ms [11,20,21,33,34]	Other [33]	-
Heart rate	<250 ms [11,20,21,33,34]	Other [33]	-
Respiration rate	<250 ms [11,20,21,33,34]	Other [33]	-
ECG	<250 ms [11,20,21,33,34]	Other [33]	-
EEG	<250 ms [11,20,21,33,34]	Other [33]	-
EMG	<250 ms [11,20,21,33,34]	Other [33]	-
Force	3–10 ms [20,21]	Experiment [37]	-
	1–10 ms [30]	Other [30]	200 km
	3–60 ms [28]	Experiment [39]	≈3200 miles
	<50 ms [29,35]	Experiment [40] & Simulation [35]	few hundred meters
	40 ms [29]	Experiment & Simulation [29]	-
	<100 ms [36]	Experiment [36]	-
Vibration	<5.5 ms [20,21,28,31]	Experiment [37]	-
	<50 ms [29]	Experiment [40]	few hundred meters
	1–10 ms [30]	Other [30]	200 km

**Table A2:** Jitter requirements for telesurgery.

Data Type	Reported Jitter	Source
2D camera flow	3–30 ms [11,20]	Simulation [41] Simulation[38]
3D camera flow	3–30 ms [11,20]	Simulation [41] Simulation [38]
	3–55 ms [48]	Simulation [48]
	<30 ms [28,29,30,34,38,41]	Other [30]
Audio flow	<30 ms [11,20,28,29,34]	Simulation [41] Simulation [38]
	50 ms [30]	Other [30]
	3–55 ms [48]	Simulation [48]
Force	<2 ms [11,20,29,34]	Experiment [40] Simulation [41]
	10 ms [30]	Other [30]
	1–10 ms [28]	Experiment [42]
Vibration	<2 ms [11,20,29,34]	Experiment [40] Simulation [41]
	10 ms [30]	Other [30]
	1–10 ms [28]	Experiment [42]

**Table A3:** Data rate requirements for telesurgery.

Data Type	Reported Data Rate	Source
2D camera flow	<10 Mbps [20,21]	Simulation [41] Experiment [40]
3D camera flow	137 Mbps–1.6 Gbps [20,21]	Simulation [28]
	≈8 Mbps [196]	Experiment [196]
	95–106 Mbps [197]	Experiment [197]
	2.5–5 Mbps [28,29]	Simulation [41] Experiment [40]
	1 Gbps [30]	Other [30]
	>1 Gbps [11]	Simulation [28]
Audio flow	22–200 Kbps [20,21,28,29]	Experiment [31]
Temperature	<10 Kbps [20,21,34]	Other [33]
Blood pressure	<10 Kbps [20,21,34]	Other [33]
Heart rate	<10 Kbps [20,21,34]	Other [33]
Respiration rate	<10 Kbps [20,21,34]	Other [33]
ECG	72 Kbps [20,21,34]	Other [33]
EEG	84.6 Kbps [20,21,34]	Other [33]
EMG	1.536 Mbps [20,21,34]	Other [33]
Force	128–400 Kbps [20,21]	Experiment [28,31]
	500 Kbps–1 Mbps [29]	Simulation [41]
	128 Kbps [28]	Experiment [43]
Vibration	128–400 Kbps [20]	Experiment [28,31]
	500 Kbps–1 Mbps [29]	Simulation [41]
	128 Kbps [28]	Experiment [43]

**Table A4:** Packet loss or bit error rate for telesurgery.

Data Type	Reported Loss	Source
2D camera flow	<$10^{-3}$ [20,21]	Experiment [40,41]
3D camera flow	<$10^{-3}$ [20,21]	Experiments [40,41]
	<1% [28,29]	Experiments [40,41] & Simulation [38]
	0.01–0.06% [48]	Simulations [48]
Audio flow	<$10^{-2}$ [20,21]	Experiments [40,41]
	0.01–0.06% [48]	Simulations [48]
	<1% [28,29]	Experiments [40,41], Simulation [38]
	$10^{-5}$ [30]	Other [30]
Temperature	<$10^{-3}$ [20,21]	Other [33]
	<$10^{-10}$ [34] (BER)	Other [33]
Blood pressure	<$10^{-3}$ [20,21]	Other [33]
	<$10^{-10}$ [34] (BER)	Other [33]
Heart rate	<$10^{-3}$ [20,21]	Other [33]
	<$10^{-10}$ [34] (BER)	Other [33]
Respiration rate	<$10^{-3}$ [20,21]	Other [33]
	<$10^{-10}$ [34] (BER)	Other [33]
ECG	<$10^{-3}$ [20,21]	Other [33]
	<$10^{-10}$ [34] (BER)	Other [33]
EEG	<$10^{-3}$ [20,21]	Other [33]
	<$10^{-10}$ [34] (BER)	Other [33]
EMG	<$10^{-3}$ [20,21]	Other [33]
	<$10^{-10}$ [34] (BER)	Other [33]
Force	<10% [29]	Experiments [40,41]
	<$10^{-4}$ [20] [21]	Experiments [40,41]
	0.01-10% [28]	Experiments [40,41]
	<0.1 [35]	Experiments [35]
Vibration	<10% [29]	Experiments [40,41,43]
	<$10^{-4}$ [20] [21]	Experiments [40,41,43]
	0.01–10% [28]	Experiments [40,41,43]

**Table A5:** Other requirements for telesurgery.

KPI	Reported Requirement	Source
Reliability	$1-10^{-7}$	[11,44]
Availability	$1-10^{-5}$	[11]
Payload size	Big	[11]
Traffic density	Low [$\mathrm{Gbps}/{\mathrm{km}}^{2}$]	[11]
Connection density	Low [/km${}^{2}$]	[11]
Service area dimension	10 m × 10 m × 5 m	[11]
Survival time	0 ms	[11]
Range	Up to 200 km	[30]
	300 km	[11]
Duty cycle for vital signal monitoring	<1–10%	[34]

## References

[1] Li D. (2019). 5G and intelligence medicine—How the next generation of wireless technology will reconstruct healthcare?. Precis. Clin. Med..

[2] Liu E., Effiok E., Hitchcock J. (2020). Survey on health care applications in 5G networks. IET Commun..

[3] Hamm S., Schleser A.C., Hartig J., Thomas P., Zoesch S., Bulitta C. (2020). 5G as enabler for Digital Healthcare. Curr. Dir. Biomed. Eng..

[4] Padmashree T., Nayak S.S. 5G Technology for E-Health. Proceedings of the 2020 Fourth International Conference on I-SMAC (IoT in Social, Mobile, Analytics and Cloud)(I-SMAC).

[5] Gupta P., Ghosh M. (2019). Revolutionizing Healthcare with 5G. Telecom Bus. Rev..

[6] Qureshi H.N., Manalastas M., Zaidi S.M.A., Imran A., Al Kalaa M.O. (2020). Service Level Agreements for 5G and beyond: Overview, Challenges and Enablers of 5G-Healthcare Systems. IEEE Access.

[7] Ullah H., Nair N.G., Moore A., Nugent C., Muschamp P., Cuevas M. (2019). 5G communication: An overview of vehicle-to-everything, drones, and healthcare use-cases. IEEE Access.

[8] Muzammil S. (2020). Telehealth: Is It Only for the Rural Areas? A Review of Its Wider Use. Telehealth Med. Today.

[9] Qadri Y.A., Nauman A., Zikria Y.B., Vasilakos A.V., Kim S.W. (2020). The future of healthcare internet of things: A survey of emerging technologies. IEEE Commun. Surv. Tutor..

[10] FDA Radio Frequency Wireless Technology in Medical Devices, Guidance for Industry and Food and Drug Administration Staff. https://www.fda.gov/media/71975/download.

[11] Cisotto G., Casarin E., Tomasin S. (2020). Requirements and Enablers of Advanced Healthcare Services over Future Cellular Systems. IEEE Commun. Mag..

[12] Schaich F., Hamon M.H., Hunukumbure M., Lorca J., Pedersen K., Schubert M., Kosmatos E., Wunder G., Reaz K. The ONE5G Approach Towards the Challenges of Multi-Service Operation in 5G Systems. Proceedings of the 2018 IEEE 87th Vehicular Technology Conference (VTC Spring).

[13] 5GPPP White Paper on Service Performance Measurement Methods over 5G Experimental Networks from TMV WG. https://5g-ppp.eu/white-paper-on-service-performance-measurement-methods-over-5g-experimental-networks/.

[14] 5G-Monarch Documentation of Requirements and KPIs and Definition of Suitable Evaluation Criteria. https://5g-monarch.eu/wp-content/uploads/2017/10/5G-MoNArch_761445_D6.1_Documentation_of_Requirements_and_KPIs_and_Definition_of_Suitable_Evaluation_Criteria_v1.0.pdf.

[15] Krasniqi F., Gavrilovska L., Maraj A., Poulkov V. (2019). The Analysis of Key Performance Indicators (KPI) in 4G/LTE Networks. Future Access Enablers for Ubiquitous and Intelligent Infrastructures.

[16] 3GPP (2017). 3GPP TR 38.913, “Study on Scenarios and Requirements for Next Generation Access Technologies”. http://www.3gpp.org.

[17] Dwivedi S., Shreevastav R., Munier F., Nygren J., Siomina I., Lyazidi Y., Shrestha D., Lindmark G., Ernström P., Stare E. (2021). Positioning in 5G networks. arXiv.

[18] Gutierrez-Estevez D.M., Gramaglia M., De Domenico A., Di Pietro N., Khatibi S., Shah K., Tsolkas D., Arnold P., Serrano P. The path towards resource elasticity for 5G network architecture. Proceedings of the 2018 IEEE Wireless Communications and Networking Conference Workshops (WCNCW).

[19] Global Connected Wearable Devices. https://www.statista.com/statistics/487291/global-connected-wearable-devices/.

[20] Zhang Q., Liu J., Zhao G. (2018). Towards 5G enabled tactile robotic telesurgery. arXiv.

[21] Usman M.A., Philip N.Y., Politis C. 5G enabled mobile healthcare for ambulances. Proceedings of the 2019 IEEE Globecom Workshops (GC Wkshps).

[22] Thuemmler C., Gavrasm A., Jumelle A., Paulin A., Sadique A., Schneider A., Fedell C., Abraham D., Trossen D. (2015). 5G and e-Health; 5G-PPP White Paper. https://5g-ppp.eu/euro-5g/.

[23] Fabrlzio M.D., Lee B.R., Chan D.Y., Stoianovici D., Jarrett T.W., Yang C., Kavoussi L.R. (2000). Effect of time delay on surgical performance during telesurgical manipulation. J. Endourol..

[24] Rayman R., Primak S., Patel R., Moallem M., Morady R., Tavakoli M., Subotic V., Galbraith N., Van Wynsberghe A., Croome K. (2005). Effects of latency on telesurgery: An experimental study. Lecture Notes in Computer Science, Proceedings of the International Conference on Medical Image Computing and Computer-Assisted Intervention, Palm Springs, CA, USA, 26–29 October 2005.

[25] Marescaux J., Leroy J., Rubino F., Smith M., Vix M., Simone M., Mutter D. (2002). Transcontinental robot-assisted remote telesurgery: Feasibility and potential applications. Ann. Surg..

[26] Xu S., Perez M., Yang K., Perrenot C., Felblinger J., Hubert J. (2014). Determination of the latency effects on surgical performance and the acceptable latency levels in telesurgery using the dV-Trainer^®^ simulator. Surg. Endosc..

[27] Perez M., Xu S., Chauhan S., Tanaka A., Simpson K., Abdul-Muhsin H., Smith R. (2016). Impact of delay on telesurgical performance: Study on the robotic simulator dV-Trainer. Int. J. Comput. Assist. Radiol. Surg..

[28] Eid M., Cha J., El Saddik A. (2010). Admux: An adaptive multiplexer for haptic-audio-visual data communication. IEEE Trans. Instrum. Meas..

[29] Marshall A., Yap K.M., Yu W. (2008). Providing QoS for networked peers in distributed haptic virtual environments. Adv. Multimed..

[30] NSF (2016). NSF Follow-on Workshop on Ultra-Low Latency Wireless Networks. NSF Workshop on Ultra Low-Latency Wireless Networks.

[31] Cizmeci B., Xu X., Chaudhari R., Bachhuber C., Alt N., Steinbach E. (2017). A multiplexing scheme for multimodal teleoperation. ACM Trans. Multimed. Comput. Commun. Appl. (TOMM).

[32] Marescaux J., Leroy J., Gagner M., Rubino F., Mutter D., Vix M., Butner S.E., Smith M.K. (2001). Transatlantic robot-assisted telesurgery. Nature.

[33] Zhen B., Patel M., Lee S., Won E., Astrin A. (2008). TG6 technical requirements document (TRD). IEEE P802.

[34] Patel M., Wang J. (2010). Applications, challenges, and prospective in emerging body area networking technologies. IEEE Wirel. Commun..

[35] Shi Z., Zou H., Rank M., Chen L., Hirche S., Muller H.J. (2009). Effects of packet loss and latency on the temporal discrimination of visual-haptic events. IEEE Trans. Haptics.

[36] Makino Y., Furuyama Y., Inoue S., Shinoda H. HaptoClone (Haptic-Optical Clone) for Mutual Tele-Environment by Real-time 3D Image Transfer with Midair Force Feedback. Proceedings of the CHI.

[37] Hachisu T., Kajimoto H. (2016). Vibration feedback latency affects material perception during rod tapping interactions. IEEE Trans. Haptics.

[38] Bertsekas D.P. (2005). Traffic Behavior and Queuing in a QoS Environment. https://www.cpe.ku.ac.th/~anan/myhomepage/wp-content/uploads/2015/01/1-opnet_full_presentation.pdf.

[39] Kim J., Kim H., Tay B.K., Muniyandi M., Srinivasan M.A., Jordan J., Mortensen J., Oliveira M., Slater M. (2004). Transatlantic touch: A study of haptic collaboration over long distance. Presence Teleoperators Virtual Environ..

[40] Souayed R.T., Gaiti D., Yu W., Dodds G., Marshall A. Experimental study of haptic interaction in distributed virtual environments. Proceedings of the EuroHaptics.

[41] Yap K.M., Marshall A., Yu W., Dodds G., Gu Q., Souayed R.T. (2005). Characterising distributed haptic virtual environment network traffic flows. IFIP—The International Federation for Information Processing, Proceedings of the International Conference on Network Control and Engineering for QoS, Security and Mobility, Lannion, France, 14–18 November 2005.

[42] Park K.S., Kenyon R.V. Effects of network characteristics on human performance in a collaborative virtual environment. Proceedings of the IEEE Virtual Reality (Cat. No. 99CB36316).

[43] Dev P., Harris D., Gutierrez D., Shah A., Senger S. (2002). End-to-end performance measurement of Internet based medical applications. Proceedings of the AMIA Symposium.

[44] Soldani D., Fadini F., Rasanen H., Duran J., Niemela T., Chandramouli D., Hoglund T., Doppler K., Himanen T., Laiho J. 5G mobile systems for healthcare. Proceedings of the 2017 IEEE 85th Vehicular Technology Conference (VTC Spring).

[45] Xia S.B., Lu Q.S. (2021). Development status of telesurgery robotic system. Chin. J. Traumatol..

[46] Valdez L.B., Datta R.R., Babic B., Müller D.T., Bruns C.J., Fuchs H.F. (2021). 5G mobile communication applications for surgery: An overview of the latest literature. Artif. Intell. Gastrointest. Endosc..

[47] Dohler M. (2021). The Internet of Skills: How 5G-Synchronized Reality Is Transforming Robotic Surgery. Robotic Surgery.

[48] Sedaghat S., Jahangir A.H. (2021). RT-TelSurg: Real Time Telesurgery Using SDN, Fog, and Cloud as Infrastructures. IEEE Access.

[49] Ahvar E., Ahvar S., Raza S.M., Manuel Sanchez Vilchez J., Lee G.M. (2021). Next generation of SDN in cloud-fog for 5G and beyond-enabled applications: Opportunities and challenges. Network.

[50] Aggarwal S., Kumar N. (2019). Fog computing for 5G-enabled tactile Internet: Research issues, challenges, and future research directions. Mob. Netw. Appl..

[51] Hartmann M., Hashmi U.S., Imran A. (2019). Edge computing in smart health care systems: Review, challenges, and research directions. Trans. Emerg. Telecommun. Technol..

[52] Akrivopoulos O., Chatzigiannakis I., Tselios C., Antoniou A. On the deployment of healthcare applications over fog computing infrastructure. Proceedings of the 2017 IEEE 41st Annual Computer Software and Applications Conference (COMPSAC).

[53] Mutlag A.A., Abd Ghani M.K., Arunkumar N.A., Mohammed M.A., Mohd O. (2019). Enabling technologies for fog computing in healthcare IoT systems. Future Gener. Comput. Syst..

[54] LaMonte M.P., Cullen J., Gagliano D.M., Gunawardane R., Hu P., Mackenzie C., Xiao Y. (2000). TeleBAT: Mobile telemedicine for the Brain Attack Team. J. Stroke Cerebrovasc. Dis..

[55] Terkelsen C., Nørgaard B., Lassen J., Gerdes J., Ankersen J., Rømer F., Nielsen T., Andersen H. (2002). Telemedicine used for remote prehospital diagnosing in patients suspected of acute myocardial infarction. J. Intern. Med..

[56] Hsieh J.C., Lin B.X., Wu F.R., Chang P.C., Tsuei Y.W., Yang C.C. (2010). Ambulance 12-lead electrocardiography transmission via cell phone technology to cardiologists. Telemed. E-Health.

[57] Liman T.G., Winter B., Waldschmidt C., Zerbe N., Hufnagl P., Audebert H.J., Endres M. (2012). Telestroke ambulances in prehospital stroke management: Concept and pilot feasibility study. Stroke.

[58] Yperzeele L., Van Hooff R.J., De Smedt A., Espinoza A.V., Van Dyck R., Van de Casseye R., Convents A., Hubloue I., Lauwaert D., De Keyser J. (2014). Feasibility of AmbulanCe-Based Telemedicine (FACT) study: Safety, feasibility and reliability of third generation in-ambulance telemedicine. PLoS ONE.

[59] Wu T.C., Nguyen C., Ankrom C., Yang J., Persse D., Vahidy F., Grotta J.C., Savitz S.I. (2014). Prehospital utility of rapid stroke evaluation using in-ambulance telemedicine: A pilot feasibility study. Stroke.

[60] Espinoza A.V., Van Hooff R.J., De Smedt A., Moens M., Yperzeele L., Nieboer K., Hubloue I., de Keyser J., Convents A., Tellez H.F. (2016). Development and pilot testing of 24/7 in-ambulance telemedicine for acute stroke: Prehospital stroke study at the Universitair Ziekenhuis Brussel-Project. Cerebrovasc. Dis..

[61] Itrat A., Taqui A., Cerejo R., Briggs F., Cho S.M., Organek N., Reimer A.P., Winners S., Rasmussen P., Hussain M.S. (2016). Telemedicine in prehospital stroke evaluation and thrombolysis: Taking stroke treatment to the doorstep. JAMA Neurol..

[62] Antoniou Z.C., Panayides A.S., Pantzaris M., Constantinides A.G., Pattichis C.S., Pattichis M.S. (2017). Real-time adaptation to time-varying constraints for medical video communications. IEEE J. Biomed. Health Inform..

[63] Rehman I.U., Nasralla M.M., Ali A., Philip N. Small cell-based ambulance scenario for medical video streaming: A 5G-health use case. Proceedings of the 2018 15th International Conference on Smart Cities: Improving Quality of Life Using ICT & IoT (HONET-ICT).

[64] Wang Q., Alcaraz-Calero J., Ricart-Sanchez R., Weiss M.B., Gavras A., Nikaein N., Vasilakos X., Giacomo B., Pietro G., Roddy M. (2019). Enable advanced QoS-aware network slicing in 5G networks for slice-based media use cases. IEEE Trans. Broadcast..

[65] Roddy M., Truong T., Walsh P., Al Bado M., Wu Y., Healy M., Ahearne S. 5G Network Slicing for Mission-critical use cases. Proceedings of the 2019 IEEE 2nd 5G World Forum (5GWF).

[66] Kamal M.D., Tahir A., Kamal M.B., Naeem M.A. (2020). Future Location Prediction for Emergency Vehicles Using Big Data: A Case Study of Healthcare Engineering. J. Healthc. Eng..

[67] Yu S., Yi F., Qiulin X., Liya S. A Framework of 5G Mobile-health Services for Ambulances. Proceedings of the 2020 IEEE 20th International Conference on Communication Technology (ICCT).

[68] Bin-Yahya M.A.R. (2015). E-AMBULANCE: A Real-Time Integration Platform for Heterogeneous Medical Telemetry System of Smart Ambulances. Ph.D. Thesis.

[69] Ehrler F., Siebert J.N. (2020). PedAMINES: A disruptive mHealth app to tackle paediatric medication errors. Swiss Med. Wkly..

[70] Almadani B., Bin-Yahya M., Shakshuki E.M. (2015). E-AMBULANCE: Real-time integration platform for heterogeneous medical telemetry system. Procedia Comput. Sci..

[71] Lippman J.M., Smith S.N.C., McMurry T.L., Sutton Z.G., Gunnell B.S., Cote J., Perina D.G., Cattell-Gordon D.C., Rheuban K.S., Solenski N.J. (2016). Mobile telestroke during ambulance transport is feasible in a rural EMS setting: The iTREAT Study. Telemed. e-Health.

[72] Kim H., Kim S.W., Park E., Kim J.H., Chang H. (2020). The role of fifth-generation mobile technology in prehospital emergency care: An opportunity to support paramedics. Health Policy Technol..

[73] Zhai Y., Xu X., Chen B., Lu H., Wang Y., Li S., Shi X., Wang W., Shang L., Zhao J. (2021). 5G-Network-Enabled Smart Ambulance: Architecture, Application, and Evaluation. IEEE Netw..

[74] Geisler F., Kunz A., Winter B., Rozanski M., Waldschmidt C., Weber J.E., Wendt M., Zieschang K., Ebinger M., Audebert H.J. (2019). Telemedicine in prehospital acute stroke care. J. Am. Heart Assoc..

[75] Kandimalla J., Vellipuram A.R., Rodriguez G., Maud A., Cruz-Flores S., Khatri R. (2021). Role of Telemedicine in Prehospital Stroke Care. Curr. Cardiol. Rep..

[76] Rajan S.S., Baraniuk S., Parker S., Wu T.C., Bowry R., Grotta J.C. (2015). Implementing a mobile stroke unit program in the United States: Why, how, and how much?. JAMA Neurol..

[77] Wu T.C., Parker S.A., Jagolino A., Yamal J.M., Bowry R., Thomas A., Yu A., Grotta J.C. (2017). Telemedicine can replace the neurologist on a mobile stroke unit. Stroke.

[78] Audebert H., Fassbender K., Hussain M.S., Ebinger M., Turc G., Uchino K., Davis S., Alexandrov A., Grotta J. (2017). The PRE-hospital stroke treatment organization. Int. J. Stroke.

[79] EU 5G PPP Trials Working Group (Including J. Alcaraz Calero and Q. Wang) The 5G PPP Infrastructure-Trials and Pilots Brochure. https://5g-ppp.eu/wp-content/uploads/2019/09/5GInfraPPP_10TPs_Brochure_FINAL_low_singlepages.pdf.

[80] Martinez-Alpiste I., Jose M., Alcaraz C., Qi W., Gelayol G., Chirivella-Perez E., Salva-Garcia P. 5G Can Shape Mission-Critical Healthcare Services. https://https://www.comsoc.org/publications/ctn/5g-can-shape-mission-critical-healthcare-services.

[81] MIoT Internet of Medical Things Revolutionizing Healthcare. https://aabme.asme.org/posts/internet-of-medical-things-revolutionizing-healthcare.

[82] Lukowicz P., Anliker U., Ward J., Troster G., Hirt E., Neufelt C. AMON: A wearable medical computer for high risk patients. Proceedings of the Sixth International Symposium on Wearable Computers.

[83] Diaz K.M., Krupka D.J., Chang M.J., Peacock J., Ma Y., Goldsmith J., Schwartz J.E., Davidson K.W. (2015). Fitbit^®^: An accurate and reliable device for wireless physical activity tracking. Int. J. Cardiol..

[84] Reeder B., David A. (2016). Health at hand: A systematic review of smart watch uses for health and wellness. J. Biomed. Inform..

[85] Trung T.Q., Ramasundaram S., Hwang B.U., Lee N.E. (2016). An all-elastomeric transparent and stretchable temperature sensor for body-attachable wearable electronics. Adv. Mater..

[86] Yamamoto Y., Yamamoto D., Takada M., Naito H., Arie T., Akita S., Takei K. (2017). Efficient skin temperature sensor and stable gel-less sticky ECG sensor for a wearable flexible healthcare patch. Adv. Healthc. Mater..

[87] Adiputra R., Hadiyoso S., Hariyani Y.S. (2018). Internet of things: Low cost and wearable SpO2 device for health monitoring. Int. J. Electr. Comput. Eng..

[88] Azhari A., Yoshimoto S., Nezu T., Iida H., Ota H., Noda Y., Araki T., Uemura T., Sekitani T., Morii K. A patch-type wireless forehead pulse oximeter for SpO_2_ measurement. Proceedings of the 2017 IEEE Biomedical Circuits and Systems Conference (BioCAS).

[89] Chacon P.J., Pu L., da Costa T.H., Shin Y.H., Ghomian T., Shamkhalichenar H., Wu H.C., Irving B.A., Choi J.W. (2018). A wearable pulse oximeter with wireless communication and motion artifact tailoring for continuous use. IEEE Trans. Biomed. Eng..

[90] Surrel G., Rincón F., Murali S., Atienza D. Low-power wearable system for real-time screening of obstructive sleep apnea. Proceedings of the 2016 IEEE Computer Society Annual Symposium on VLSI (ISVLSI).

[91] Shilaih M., Goodale B.M., Falco L., Kübler F., De Clerck V., Leeners B. (2018). Modern fertility awareness methods: Wrist wearables capture the changes in temperature associated with the menstrual cycle. Biosci. Rep..

[92] Xie R., Du Q., Zou B., Chen Y., Zhang K., Liu Y., Liang J., Zheng B., Li S., Zhang W. (2019). Wearable leather-based electronics for respiration monitoring. ACS Appl. Bio Mater..

[93] Mizuno A., Changolkar S., Patel M.S. (2020). Wearable Devices to Monitor and Reduce the Risk of Cardiovascular Disease: Evidence and Opportunities. Annu. Rev. Med..

[94] Holz C., Wang E.J. (2017). Glabella: Continuously sensing blood pressure behavior using an unobtrusive wearable device. Proc. ACM Interactive Mobile Wearable Ubiquitous Technol..

[95] Kuwabara M., Harada K., Hishiki Y., Kario K. (2019). Validation of two watch-type wearable blood pressure monitors according to the ANSI/AAMI/ISO81060-2: 2013 guidelines: Omron HEM-6410T-ZM and HEM-6410T-ZL. J. Clin. Hypertens..

[96] Arakawa T. (2018). Recent research and developing trends of wearable sensors for detecting blood pressure. Sensors.

[97] Escobedo P., Ramos-Lorente C.E., Martínez-Olmos A., Carvajal M.A., Ortega-Munoz M., de Orbe-Paya I., Hernández-Mateo F., Santoyo-González F., Capitán-Vallvey L.F., Palma A.J. (2021). Wireless wearable wristband for continuous sweat pH monitoring. Sens. Actuators B Chem..

[98] Nakata S., Shiomi M., Fujita Y., Arie T., Akita S., Takei K. (2018). A wearable pH sensor with high sensitivity based on a flexible charge-coupled device. Nat. Electron..

[99] Wijsman J., Grundlehner B., Liu H., Hermens H., Penders J. Towards mental stress detection using wearable physiological sensors. Proceedings of the 2011 Annual International Conference of the IEEE Engineering in Medicine and Biology Society.

[100] Valenza G., Nardelli M., Lanata A., Gentili C., Bertschy G., Paradiso R., Scilingo E.P. (2013). Wearable monitoring for mood recognition in bipolar disorder based on history-dependent long-term heart rate variability analysis. IEEE J. Biomed. Health Inform..

[101] Gruwez A., Bruyneel A.V., Bruyneel M. (2019). The validity of two commercially-available sleep trackers and actigraphy for assessment of sleep parameters in obstructive sleep apnea patients. PLoS ONE.

[102] Lin C.T., Ko L.W., Chang M.H., Duann J.R., Chen J.Y., Su T.P., Jung T.P. (2010). Review of wireless and wearable electroencephalogram systems and brain-computer interfaces–a mini-review. Gerontology.

[103] Casson A.J., Yates D.C., Smith S.J., Duncan J.S., Rodriguez-Villegas E. (2010). Wearable electroencephalography. IEEE Eng. Med. Biol. Mag..

[104] Ip J.E. (2019). Wearable devices for cardiac rhythm diagnosis and management. JAMA.

[105] Jeon B., Lee J., Choi J. (2013). Design and implementation of a wearable ECG system. Int. J. Smart Home.

[106] Beniczky S., Conradsen I., Henning O., Fabricius M., Wolf P. (2018). Automated real-time detection of tonic-clonic seizures using a wearable EMG device. Neurology.

[107] Tsubouchi Y., Suzuki K. BioTones: A wearable device for EMG auditory biofeedback. Proceedings of the 2010 Annual International Conference of the IEEE Engineering in Medicine and Biology.

[108] Nathan V., Jafari R. (2017). Particle filtering and sensor fusion for robust heart rate monitoring using wearable sensors. IEEE J. Biomed. Health Inform..

[109] Park J.H., Jang D.G., Park J.W., Youm S.K. (2015). Wearable sensing of in-ear pressure for heart rate monitoring with a piezoelectric sensor. Sensors.

[110] El-Amrawy F., Nounou M.I. (2015). Are currently available wearable devices for activity tracking and heart rate monitoring accurate, precise, and medically beneficial?. Healthc. Inform. Res..

[111] Tsai C.W., Li C.H., Lam R.W.K., Li C.K., Ho S. (2019). Diabetes care in motion: Blood glucose estimation using wearable devices. IEEE Consum. Electron. Mag..

[112] Cappon G., Acciaroli G., Vettoretti M., Facchinetti A., Sparacino G. (2017). Wearable continuous glucose monitoring sensors: A revolution in diabetes treatment. Electronics.

[113] Pickup J.C. (2012). Insulin-pump therapy for type 1 diabetes mellitus. N. Engl. J. Med..

[114] Weissberg-Benchell J., Antisdel-Lomaglio J., Seshadri R. (2003). Insulin pump therapy: A meta-analysis. Diabetes Care.

[115] Gadaleta M., Facchinetti A., Grisan E., Rossi M. (2018). Prediction of adverse glycemic events from continuous glucose monitoring signal. IEEE J. Biomed. Health Inform..

[116] Angelucci A., Kuller D., Aliverti A. (2020). A home telemedicine system for continuous respiratory monitoring. IEEE J. Biomed. Health Inform..

[117] Scherer M., Menachery K., Magno M. SmartAid: A Low-Power Smart Hearing Aid For Stutterers. Proceedings of the 2019 IEEE Sensors Applications Symposium (SAS).

[118] Sudharsan B., Chockalingam M. (2019). A microphone array and voice algorithm based smart hearing aid. arXiv.

[119] DJordjevic S., Stancin S., Meglc A., Milutinovic V., Tomazic S. (2011). Mc sensor—A novel method for measurement of muscle tension. Sensors.

[120] Mansuri B., Torabinejhad F., Jamshidi A.A., Dabirmoghaddam P., Vasaghi-Gharamaleki B., Ghelichi L. (2020). Transcutaneous electrical nerve stimulation combined with voice therapy in women with muscle tension dysphonia. J. Voice.

[121] Velázquez R. (2010). Wearable assistive devices for the blind. Wearable and Autonomous Biomedical Devices and Systems for Smart Environment.

[122] Garcia-Macias J.A., Ramos A.G., Hasimoto-Beltran R., Hernandez S.E.P. (2019). Uasisi: A modular and adaptable wearable system to assist the visually impaired. Procedia Comput. Sci..

[123] Savindu H.P., Iroshan K., Panangala C.D., Perera W., De Silva A.C. BrailleBand: Blind support haptic wearable band for communication using braille language. Proceedings of the 2017 IEEE International Conference on Systems, Man, and Cybernetics (SMC).

[124] Sun M., Burke L.E., Mao Z.H., Chen Y., Chen H.C., Bai Y., Li Y., Li C., Jia W. eButton: A wearable computer for health monitoring and personal assistance. Proceedings of the 51st Annual Design Automation Conference.

[125] Kapur A., Kapur S., Maes P. Alterego: A personalized wearable silent speech interface. Proceedings of the 23rd International Conference on Intelligent User Interfaces.

[126] Marjanovic N., Piccinini G., Kerr K., Esmailbeigi H. TongueToSpeech (TTS): Wearable wireless assistive device for augmented speech. Proceedings of the 2017 39th Annual International Conference of the IEEE Engineering in Medicine and Biology Society (EMBC).

[127] Huo W., Mohammed S., Moreno J.C., Amirat Y. (2014). Lower limb wearable robots for assistance and rehabilitation: A state of the art. IEEE Syst. J..

[128] Hadi A., Alipour K., Kazeminasab S., Elahinia M. (2018). ASR glove: A wearable glove for hand assistance and rehabilitation using shape memory alloys. J. Intell. Mater. Syst. Struct..

[129] Gandolla M., Antonietti A., Longatelli V., Pedrocchi A. (2020). The effectiveness of wearable upper limb assistive devices in degenerative neuromuscular diseases: A systematic review and meta-analysis. Front. Bioeng. Biotechnol..

[130] Chen B., Zhong C.H., Zhao X., Ma H., Guan X., Li X., Liang F.Y., Cheng J.C.Y., Qin L., Law S.W. (2017). A wearable exoskeleton suit for motion assistance to paralysed patients. J. Orthop. Transl..

[131] Delahoz Y.S., Labrador M.A. (2014). Survey on fall detection and fall prevention using wearable and external sensors. Sensors.

[132] Chen D., Feng W., Zhang Y., Li X., Wang T. A wearable wireless fall detection system with accelerators. Proceedings of the 2011 IEEE International Conference on Robotics and Biomimetics.

[133] Yi W.J., Saniie J. Design flow of a wearable system for body posture assessment and fall detection with android smartphone. Proceedings of the 2014 IEEE International Technology Management Conference.

[134] Bruno E., Biondi A., Thorpe S., Richardson M., Consortium R.C. (2020). Patients self-mastery of wearable devices for seizure detection: A direct user-experience. Seizure.

[135] Jeppesen J., Fuglsang-Frederiksen A., Johansen P., Christensen J., Wüstenhagen S., Tankisi H., Qerama E., Hess A., Beniczky S. (2019). Seizure detection based on heart rate variability using a wearable electrocardiography device. Epilepsia.

[136] Pierleoni P., Belli A., Palma L., Pellegrini M., Pernini L., Valenti S. (2015). A high reliability wearable device for elderly fall detection. IEEE Sens. J..

[137] Atallah L., Lo B., King R., Yang G.Z. (2011). Sensor positioning for activity recognition using wearable accelerometers. IEEE Trans. Biomed. Circuits Syst..

[138] Ouyang H., Liu Z., Li N., Shi B., Zou Y., Xie F., Ma Y., Li Z., Li H., Zheng Q. (2019). Symbiotic cardiac pacemaker. Nat. Commun..

[139] Eicken A., Kolb C., Lange S., Brodherr-Heberlein S., Zrenner B., Schreiber C., Hess J. (2006). Implantable cardioverter defibrillator (ICD) in children. Int. J. Cardiol..

[140] Van der Kroft S. (2021). Design and Validation of an Implantable Actuator for Use in a Novel Dynamic Arteriovenous Shunt System. Master’s Thesis.

[141] Shiba K., Tsuji T., Koshiji K. (2006). Direct drive of an implantable actuator using a transcutaneous energy transmission system. J. Life Support Eng..

[142] (2008). BAN Applications Matrix, Document 15-07-0735-08-0. https://www.ieee802.org/15/pub/default_page.html.

[143] Rong G., Zheng Y., Sawan M. (2021). Energy Solutions for Wearable Sensors: A Review. Sensors.

[144] Kos A., Milutinović V., Umek A. (2019). Challenges in wireless communication for connected sensors and wearable devices used in sport biofeedback applications. Future Gener. Comput. Syst..

[145] Ullah S., Khan P., Ullah N., Saleem S., Higgins H., Kwak K.S. (2010). A review of wireless body area networks for medical applications. arXiv.

[146] Movassaghi S., Abolhasan M., Lipman J., Smith D., Jamalipour A. (2014). Wireless body area networks: A survey. IEEE Commun. Surv. Tutor..

[147] (2008). TG6 Applications Matrix, Document 15-08-0406-00-0006, IEEE P802. https://view.officeapps.live.com/op/view.aspx?src=https%3A%2F%2Fmentor.ieee.org%2F802.15%2Fdcn%2F08%2F15-08-0644-09-0006-tg6-technical-requirements-document.doc.

[148] Jones R.W., Katzis K. 5G and wireless body area networks. Proceedings of the 2018 IEEE Wireless Communications and Networking Conference Workshops (WCNCW).

[149] Soh P.J., Vandenbosch G.A., Mercuri M., Schreurs D.M.P. (2015). Wearable wireless health monitoring: Current developments, challenges, and future trends. IEEE Microw. Mag..

[150] Santagati G.E., Melodia T. (2016). A software-defined ultrasonic networking framework for wearable devices. IEEE/ACM Trans. Netw..

[151] Garcia-Perez C., Diaz-Zayas A., Rios A., Merino P., Katsalis K., Chang C.Y., Shariat S., Nikaein N., Rodriguez P., Morris D. (2017). Improving the efficiency and reliability of wearable based mobile eHealth applications. Pervasive Mob. Comput..

[152] Sahni Y., Cao J., Zhang S., Yang L. (2017). Edge mesh: A new paradigm to enable distributed intelligence in internet of things. IEEE Access.

[153] Gia T.N., Jiang M., Rahmani A.M., Westerlund T., Liljeberg P., Tenhunen H. Fog computing in healthcare internet of things: A case study on ecg feature extraction. Proceedings of the 2015 IEEE International Conference on Computer and INFORMATION technology; Ubiquitous Computing and Communications; Dependable, Autonomic and Secure Computing; Pervasive Intelligence and Computing.

[154] ISO standard ANSI/AAMI/ISO 14971:2019-Medical Devices-Application of Risk Management to Medical Devices. https://www.iso.org/standard/72704.html.

[155] FDA Content of Premarket Submissions for Management of Cybersecurity in Medical Devices: Draft Guidance for Industry and Food and Drug Administration Staff. https://www.fda.gov/media/119933/download.

[156] Motani M., Yap K.K., Natarajan A., de Silva B., Hu S., Chua K.C. Network characteristics of urban environments for wireless BAN. Proceedings of the 2007 IEEE Biomedical Circuits and Systems Conference.

[157] Al Kalaa M.O., Balid W., Refai H.H., LaSorte N.J., Seidman S.J., Bassen H.I., Silberberg J.L., Witters D. (2016). Characterizing the 2.4 GHz spectrum in a hospital environment: Modeling and applicability to coexistence testing of medical devices. IEEE Trans. Electromagn. Compat..

[158] Qualcomm Technologies, Inc VR and AR Pushing Connectivity Limits. https://www.qualcomm.com/media/documents/files/vr-and-ar-pushing-connectivity-limits.pdf.

[159] Pozo A.P., Toksvig M., Schrager T.F., Hsu J., Mathur U., Sorkine-Hornung A., Szeliski R., Cabral B. (2019). An integrated 6DoF video camera and system design. ACM Trans. Graph. (TOG).

[160] Feil-Seifer D., Mataric M.J. Defining socially assistive robotics. Proceedings of the 9th International Conference on Rehabilitation Robotics, 2005, ICORR 2005.

[161] Pavón-Pulido N., López-Riquelme J.A., Ferruz-Melero J., Vega-Rodríguez M.Á., Barrios-León A.J. (2014). A service robot for monitoring elderly people in the context of ambient assisted living. J. Ambient Intell. Smart Environ..

[162] Bonaccorsi M., Fiorini L., Cavallo F., Esposito R., Dario P. (2015). Design of cloud robotic services for senior citizens to improve independent living and personal health management. Ambient Assisted Living.

[163] Ma Y., Zhang Y., Wan J., Zhang D., Pan N. (2015). Robot and cloud-assisted multi-modal healthcare system. Clust. Comput..

[164] Gross H.M., Mueller S., Schroeter C., Volkhardt M., Scheidig A., Debes K., Richter K., Doering N. Robot companion for domestic health assistance: Implementation, test and case study under everyday conditions in private apartments. Proceedings of the 2015 IEEE/RSJ International Conference on Intelligent Robots and Systems (IROS).

[165] Manzi A., Fiorini L., Limosani R., Sincak P., Dario P., Cavallo F. Use case evaluation of a cloud robotics teleoperation system (short paper). Proceedings of the 2016 5th IEEE International Conference on Cloud Networking (Cloudnet).

[166] Bonaccorsi M., Fiorini L., Cavallo F., Saffiotti A., Dario P. (2016). A cloud robotics solution to improve social assistive robots for active and healthy aging. Int. J. Soc. Robot..

[167] Fiorini L., Esposito R., Bonaccorsi M., Petrazzuolo C., Saponara F., Giannantonio R., De Petris G., Dario P., Cavallo F. (2017). Enabling personalised medical support for chronic disease management through a hybrid robot-cloud approach. Auton. Robot..

[168] Cádrik T., Takáč P., Ondo J., Sinčák P., Mach M., Jakab F., Cavallo F., Bonaccorsi M. (2017). Cloud-based robots and intelligent space teleoperation tools. Robot Intelligence Technology and Applications 4.

[169] Cavallo F., Limosani R., Fiorini L., Esposito R., Furferi R., Governi L., Carfagni M. (2018). Design impact of acceptability and dependability in assisted living robotic applications. Int. J. Interact. Des. Manuf. (IJIDeM).

[170] Brunete A., Gambao E., Hernando M., Cedazo R. (2021). Smart Assistive Architecture for the Integration of IoT Devices, Robotic Systems, and Multimodal Interfaces in Healthcare Environments. Sensors.

[171] Tröbinger M., Jähne C., Qu Z., Elsner J., Reindl A., Getz S., Goll T., Loinger B., Loibl T., Kugler C. (2021). Introducing GARMI-A Service Robotics Platform to Support the Elderly at Home: Design Philosophy, System Overview and First Results. IEEE Robot. Autom. Lett..

[172] Witrisal K., Meissner P., Leitinger E., Shen Y., Gustafson C., Tufvesson F., Haneda K., Dardari D., Molisch A.F., Conti A. (2016). High-accuracy localization for assisted living: 5G systems will turn multipath channels from foe to friend. IEEE Signal Process. Mag..

[173] RADIO Project Unobtrusive, Efficient, Reliable and Modular Solutions for Independent Ageing. http://www.radio-project.eu/.

[174] Ramoly N., Bouzeghoub A., Finance B. (2018). A framework for service robots in smart home: An efficient solution for domestic healthcare. IRBM.

[175] Kaneriya S., Vora J., Tanwar S., Tyagi S. Standardising the use of duplex channels in 5G-WiFi networking for ambient assisted living. Proceedings of the 2019 IEEE International Conference on Communications Workshops (ICC Workshops).

[176] Henry S., Alsohaily A., Sousa E.S. (2020). 5G is real: Evaluating the compliance of the 3GPP 5G new radio system with the ITU IMT-2020 requirements. IEEE Access.

[177] European 5G Observatory 5G Trials That Have Been Publicly Announced in EU27, UK, Norway, Russia, Switzerland and Turkey. https://5gobservatory.eu/5g-trial/major-european-5g-trials-and-pilots/.

[178] sdx Central 5G Trials in the United States—Steps Toward Standardization. https://www.sdxcentral.com/5g/definitions/5g-trials/.

[179] Verizon Verizon Will Rapidly Integrate C-Band Spectrum with mmWave for Customers. https://www.verizon.com/about/news/verizon-c-band-spectrum-mmwave.

[180] Verizon Explore 4G LTE and 5G Network Coverage in Your Area. https://www.verizon.com/coverage-map/.

[181] AT&T Wireless Coverage. https://www.att.com/maps/wireless-coverage.html.

[182] T-Mobile Coverage Maps. https://www.t-mobile.com/coverage/coverage-map.

[183] Opensignal How AT&T, Sprint, T-Mobile and Verizon Differ in Their Early 5G Approach. https://www.opensignal.com/2020/02/20/how-att-sprint-t-mobile-and-verizon-differ-in-their-early-5g-approach.

[184] Digital Trends 5G vs. 4G: HowWill the Newest Network Improve on the Last?. https://www.digitaltrends.com/mobile/5g-vs-4g/.

[185] Forbes 5G Latency Improvements Are Still Lagging. https://www.forbes.com/sites/bobodonnell/2020/02/18/5g-latency-improvements-are-still-lagging/?sh=6d74337548f1.

[186] Carrozzo G., Siddiqui M.S., Du K., Sayadi B., Carrasco O., Lazarakis F., Sterle J., Bruschi R. Definition and Evaluation of Latency in 5G with Heterogeneous Use Cases and Architectures. https://www.5gcity.eu/wp-content/uploads/2020/05/Definition-and-Evaluation-of-Latency-in-5G-with-Heterogeneous-Use-Cases-and-Architectures.pdf.

[187] Asghar A., Farooq H., Imran A. Concurrent CCO and LB optimization in emerging HetNets: A novel solution and comparative analysis. Proceedings of the 2018 IEEE 29th Annual International Symposium on Personal, Indoor and Mobile Radio Communications (PIMRC).

[188] Qureshi H.N., Imran A. (2019). On the tradeoffs between coverage radius, altitude, and beamwidth for practical UAV deployments. IEEE Trans. Aerosp. Electron. Syst..

[189] Park S.H., Kang N.G., Cho C., Won E.T., Patro R.K., Goyal G., Bhatia A., Bynam K., Naniyat A. (2008). System Simulation Metrics for BAN—Samsung. Project: IEEE P802.15 Working Group for Wireless Personal Area Networks (WPANs). https://mentor.ieee.org/802.15/file/08/15-08-0630-00-0006-system-simulation-metrics-for-ban.ppt.

[190] Qureshi H.N., Manalastas M., Imran A., Kalaa M.O.A. (2021). Service Level Agreements for 5G-Enabled Healthcare Systems: Challenges and Considerations. IEEE Netw..

[191] Tian W., Fan M., Zeng C., Liu Y., He D., Zhang Q. (2020). Telerobotic spinal surgery based on 5G network: The first 12 cases. Neurospine.

[192] Parvez I., Rahmati A., Guvenc I., Sarwat A.I., Dai H. (2018). A survey on low latency towards 5G: RAN, core network and caching solutions. IEEE Commun. Surv. Tutor..

[193] Ron Malenfant, Cisco Industry Voices—5G Has the Potential to Transform Healthcare for Rural Communities. https://www.fiercehealthcare.com/tech/industry-voices-5g-has-potential-to-transform-healthcare-for-rural-communities.

[194] OTH Amberg-Weiden 5G4Healthcare. https://www.oth-aw.de/en/research-and-cooperation/latest-news-in-research/5g4healthcare/homepage/.

[195] Acemoglu A., Peretti G., Trimarchi M., Hysenbelli J., Krieglstein J., Geraldes A., Deshpande N., Ceysens P.M.V., Caldwell D.G., Delsanto M. (2020). Operating from a distance: Robotic vocal cord 5G telesurgery on a cadaver. Ann. Intern. Med..

[196] Jell A., Vogel T., Ostler D., Marahrens N., Wilhelm D., Samm N., Eichinger J., Weigel W., Feussner H., Friess H. (2019). 5th-Generation Mobile Communication: Data Highway for Surgery 4.0. Surg. Technol. Int..

[197] Lacy A., Bravo R., Otero-Piñeiro A., Pena R., De Lacy F., Menchaca R., Balibrea J. (2019). 5G-assisted telementored surgery. Br. J. Surg..

[198] Zheng J., Wang Y., Zhang J., Guo W., Yang X., Luo L., Jiao W., Hu X., Yu Z., Wang C. (2020). 5G ultra-remote robot-assisted laparoscopic surgery in China. Surg. Endosc..

